# Integrative analysis of chloroplast genome, chemicals, and illustrations in Bencao literature provides insights into the medicinal value of *Peucedanum huangshanense*

**DOI:** 10.3389/fpls.2023.1179915

**Published:** 2023-08-04

**Authors:** Haibing Sun, Shanshan Chu, Lu Jiang, Zhenzhen Tong, Ming’en Cheng, Huasheng Peng, Luqi Huang

**Affiliations:** ^1^ School of Pharmacy, Anhui University of Chinese Medicine, Hefei, China; ^2^ State Key Laboratory for Quality Ensurance and Sustainable Use of Dao-di Herbs, National Resource Center for Chinese Materia Medica, China Academy of Chinese Medical Sciences, Beijing, China; ^3^ Key Scientific Research Base of Traditional Chinese Medicine Heritage (Institute of Chinese Materia Medica, China Academy of Chinese Medical Sciences), State Administration of Cultural Heritage, Beijing, China; ^4^ Department of Traditional Chinese Medicine, Anhui Province Key Laboratory of Research and Development of Chinese Medicine, Hefei, China; ^5^ National Resource Center for Chinese Materia Medica, China Academy of Chinese Medical Sciences, Beijing, China

**Keywords:** *Peucedanum huangshanense*, chloroplast genome, Chinese medicine, herbal textual research, medicinal value, *Qianhu*

## Abstract

The genus *Peucedanum* L. (Apiaceae) is a large group comprising more than 120 species distributed worldwide. Many plants of the genus *Peucedanum* have been studied and used in traditional Chinese medicine. In 2020, a new species, *Peucedanum huangshanense* Lu Q. Huang, H. S. Peng & S. S. Chu, was found in the Huangshan Mountains of Anhui Province, China. However, little is known about its medicinal properties. Thus, the objective of this study is to explore the potential medicinal value of *P*. *huangshanense* and its relationship with other *Peucedanum* species. Through textual research on illustrations of *Qianhu* in Bencao literature, it can be inferred that at least five species of genus *Peucedanum* have been used in Chinese medicine. Therefore, we chose these five species of *Peucedanum* and *P*. *huangshanense* together for subsequent research. We conducted morphological, chloroplast genome, and chemical analyses of six *Peucedanum* species, including the newly discovered *P. huangshanense*. The chloroplast genomes of *Peucedanum* showed a typical tetrad structure, and the gene structure and content were similar and conservative. There were significant differences in genome size and the expansion of the inverted repeat boundary. Through nucleotide polymorphism analysis, we screened 14 hotspot mutation regions that have the potential to be used as specific molecular markers for the taxonomy of *Peucedanum*. Our results showed an inversion of the *trnD-trnY-trnE* gene in the *P. huangshanense* chloroplast genome, which can be developed as a specific molecular marker for species identification. Phylogenetic analysis showed that the phylogenetic trees had high support and resolution, which strongly supports the view that *Peucedanum* is not a monophyletic group. *P. huangshanense* had the closest genetic relationship to *P. ampliatum* K. T. Fu, followed by *P. harry-smithii* Fedde ex Wolff. Furthermore, the main coumarins of *P. huangshanense* were most similar to those of *P. japonicum* Thunb. and *P. harry-smithii*. In summary, our research lays a foundation for the systematic classification of *Peucedanum* and sheds light on the medicinal value of *P. huangshanense*.

## 1 Introduction

The genus *Peucedanum* L. (Apiaceae) has more than 120 species and grows in Europe, Asia, and Africa. Most species of the genus *Peucedanum* have important medicinal value attributed to coumarin, flavonoids, phenolic compounds, and essential fatty acids (Sarkhail, 2014). For instance, the leaf and rhizome of *P. ostruthium* (L.) W. D. J. Koch were developed to treat Alzheimer’s disease and dermatosis in central and southern Europe (Palmioli et al., 2019; Danna et al., 2022). In addition, *P. chenur* Have is an endemic species of Kurdistan in western Iran, aerial parts of *P. chenur* has inhibitory effect on human colon cancer cells (Yadegari et al., 2020). Moreover, the volatile oils in the fruits of *P. dhana* A. Ham have anti-inflammatory and antioxidant activities as well as cytotoxic effects on cancer cells; they are used to treat colon adenocarcinoma in Thailand (Khruengsai et al., 2021).

Peucedani Radix (*Qianhu* in Chinese) is a famous Chinese medicine and has a long history of medicinal use in China. *Qianhu* was first recorded in *Ming Yi Bie Lu* over 1500 years ago. *Qianhu* has the effect of down bearing Qi, resolving phlegm, dispersing wind, and clearing heat. It is commonly used to treat phlegm-heat cough, asthma, and wind-heat cough with sputum in China (Committee for the Pharmacopoeia of PR China, 2020). *Qianhu* also has medicinal records in Korea and Japan (Kanazawa et al., 2022). *Qianhu* is derived from the roots of *P. praeruptorum* Dunn and generally using the root of vegetative growth stage (not the bolting stage) (The State Pharmacopoeia Committee of China, 2020). Many species of *Peucedanum* are mixed with *P. praeruptorum* because of their similar characteristics. For example, the roots of *P. turgeniifolium* Wolff, *P. medicum* Dunn, *P. cavaleriei* H. Wolff (*Ligusticopsis brachyloba*), and *P. harry-smithii* var. *subglabrum* Shan et Sheh are still regarded as medicinal *Qianhu* in the Sichuan, Yunnan, Gansu Provinces. Therefore, it is particularly important to identify the characteristics of reproductive organs such as flowers and fruits. *P. praeruptorum* is rich in active ingredients, such as Praeruptorin A and Praeruptorin B, which are characteristic components of genuine *Qianhu* (Lee et al., 2015). These chemical components differ from those found in other species of the genus *Peucedanum*. It is a controversial issue to use the roots of other species of *Peucedanum* as substitutes for *Qianhu*. Therefore, it is important to accurately identify and differentiate common adulterants derived from the *Peucedanum* genus to ensure the safety of clinical medication.

Since the Fourth Chinese Materia Medica Resources Survey was conducted in China, more than 163 new species have been discovered. The chemical characteristics and potential medicinal value of these new species are of concern. A study of 79 new species found in the census in the “Discovery and efficacy study of new resources” showed that 60% of the new species had potential medicinal value. In 2020, *P. huangshanense* Lu Q. Huang, H. S. Peng & S. S. Chu, a new species of the genus *Peucedanum*, was discovered in Huangshan, Anhui Province, China, where the species grows on the forest margins and cliffs (elev.ca. 1600 m). Detailed morphological comparisons have shown that it is similar to *P. praeruptorum* but has larger compound umbels (5–14 cm across), rays up to 25, and long-ovoid mericarps with lateral, narrowly winged ribs (Chu et al., 2020). To explore the phylogenetic position of this species, the nuclear ribosomal DNA internal transcribed spacer (ITS) region was sequenced for *P. huangshanense*. Morphological and molecular evidence supports the hypothesis that *P. huangshanense* is a new distinct species (Chu et al., 2020). Additionally, the root of *P. huangshanense* is similar to that of *P. praeruptorum*, whether it has the similar effect is worth studying.

Since ancient times, many herbs have been regarded as the original plants of *Qianhu* and have been studied for their medicinal properties (Song et al., 2015). Additionally, the roots of some congeneric species of *P. praeruptorum* are still regarded as substitutes for *Qianhu* in clinical practice because of their similar chemical composition. Thus, further study of the similarities between *P. huangshanense* and these alternative species is warranted for the development of *P. huangshanense*. Forty species belong to the genus *Peucedanum* in China. The genus is extremely heterogeneous and exhibits great diversity in life-forms, leaf and fruit structures, and chemical constituents (Wen et al., 2021). A previous study first attempted to comprehensively investigate the plastome features and infer phylogeny using plastome data from the *Peucedanum* genus (Liu et al., 2022). The results of Liu’s research conclusively demonstrate the efficacy and power of plastome data in enhancing support and resolution within the phylogeny of *Peucedanum*. In the present study, we investigated the herbal drawings of *Qianhu* recorded in ancient and modern herbal books and records. Subsequently, we collected six medicinal species used as *Qianhu* for complete chloroplast genome and internal transcribed spacer (ITS) analysis. In addition, the contents of ten coumarins from these *Peucedanum* species were determined. Combining genetic characteristics with chemical information could speculate on the medicinal value of *P. huangshanense*. The study will promote the discovery of new medicinal resources, and is of great significance to the sustainable development of traditional Chinese medicine resources.

## 2 Materials and methods

### 2.1 Plant materials

We collected 33 accessions of genus *Peucedanum* from Anhui, Fujian, Hunan and Shanxi provinces, China, including *P. huangshanense*, *P. praeruptorum*, *P. japonicum* Thunb, *P. medicum*, *P. wawrae* (Wolff) Su and *P. harry-smithii* Fedde ex Wolff. Voucher information of the samples are shown in **Supplementary Table 1**. The morphology of six species is shown in **Supplementary Figure 1**. The fresh and healthy leaves were collected for DNA extraction, and the new chloroplast genomes generated in this paper were deposited in GenBank under accession numbers: OQ473752–OQ473757. The underground parts of samples were dried and ground into powders (50 mesh). Then powders were stored under dry conditions at room temperature before analysis.

### 2.2 DNA extraction

Total DNA was extracted from silica-dried leaves material. Genomic DNA was extracted using the Plant Genomic DNA Kit (Tiangen Biotech, Beijing, China) according to the manufacturer’s protocol. Then, 1% (w/v) agarose gel electrophoresis was used to test DNA integrity, and concentrations were determined using a NanoPhotometer® spectrophotometer (Implen, München, Germany). The extracted DNA was stored in the refrigerator at -20°C.

### 2.3 ITS amplification, sequencing and data analysis

The ITS sequence was chosen in this study, since it is one of the universal DNA barcode markers for land plants. PCR amplifications of ITS were performed using universal primer 5F (5’-GGAAGTAAAAGTCGTAACAAGG-3’) and 4R (5’-TCCTCCGCTTATTGATATGC-3’) (Zhou et al., 2014). The reaction mixture was as follows: total volume 20μL, including 2X M5 HiPer plus Taq HiFi PCR mix (with blue dye) (Mei5bio, Beijing, China) 10μL, Nuclease-free ddH_2_O 8μL, DNA template 1μL, forward primer 0.5μL and reverse primer 0.5μL. PCR program: pre-denatured at 95°C, denatured at 94°C, denatured at 10s, annealed at 55°C, extended at 72°C for 20 s, cycle 35 times, extended at 72°C for 5 min. The amplified PCR products were checked by 1% agarose gel electrophoresis to detect whether the bands were bright and single and whether the fragment size was correct. The successfully amplified PCR with bright target band were purified and sequenced at General Biology (Chuzhou, China).

ITS sequences were assembled using SeqMan v7 (Swindell and Plastere, 1997), and then aligned and manually refined in BioEdit v7.2 (Elkins, 2013). The genetic distance was conducted using the Kimura 2-parameter model (Kimura, 1980). This analysis involved 17 nucleotide sequences of *Peucedanum*. Codon positions included were 1st+2nd+3rd+Noncoding. The number of base substitutions per site from between sequences are shown. ModelFinder (Kalyaanamoorthy et al., 2017) was used to select the best-fit model using BIC criterion. Maximum likelihood (ML) phylogenetic inference was performed using IQ-TREE v1.6.8 (Nguyen et al., 2015) with 1000 bootstrap replicates based on the TIM2e+G4 nucleotide substitution model to assess branch support. Bayesian inference (BI) phylogenies were inferred using MrBayes v3.2.6 (Ronquist et al., 2012) under GTR+I+G+F model (2 parallel runs, 2,000,000 generations), in which the initial 25% of sampled data were discarded as burn-in.

### 2.4 Chloroplast genomes sequencing, assembly and annotation

Genomic DNA was fragmented into 350 bp to construct the pair-end library, and then sequenced using the Illumina NovaSeq platform at Novogene (Tianjin, China). Raw data was filtered using fastP v0.15.0 (-n 10 and -q 15) to obtain high quality reads (Chen et al., 2018). Then clean data was used to assemble the whole chloroplast genome with GetOrganelle (Jin et al, 2020) with the K-values parameter of 21, 55, 85, and 115. The chloroplast genome of *P. praeruptorum* (NC_060841.1) downloaded from the National Center for Biotechnology Information (NCBI) was used as reference. The assembled genome was annotated using CPGAVAS2 (http://47.96.249.172:16019/analyzer/home ) (Shi et al, 2019), and drew a circular physical map using OGDRAW (https://chlorobox.mpimp-golm.mpg.de/OGDraw.html) (Greiner et al., 2019).

### 2.5 Comparison of the chloroplast genomes and phylogenetic analysis

We compared the boundaries of the LSC, SSC and IR regions among the chloroplast genomes of genus Peucedanum in IRscope (Amiryousefi et al., 2018). We used MIcroSAtellite identification tool (MISA) (Mudunuri and Nagarajaram, 2007) to visualize Simple Sequence Repeat (SSRs). The parameter of MISA was set as follows: ten repeat units for mononucleotide repeat SSRs, five repeat units for dinucleotide repeat SSRs, four repeat units for trinucleotide repeat SSRs, and three repeat units for tetra-, penta-, and hexanucleotide repeat SSRs. The max length of sequence between two SSRs, registered as compound SSR, was 100 bp. The DNA rearrangements among chloroplast genomes were detected using Mauve v2.4.0 (Darling et al., 2004). Sequence divergence were investigated using the mVISTA (Frazer et al., 2004) with *P. ampliatum* (OK336475.1) as reference. The nucleotide diversity was calculated based on the sliding window using the DnaSP v5.10 (Rozas et al, 2017).

Forty-eight chloroplast genomes were used for phylogenetic analysis in this study, including 13 samples of 21 species in genus *Peucedanum* and other 27 species from Apiaceae. *Cicutavirosa* L. (KX352466) and *Cryptotaenia japonica* Hassk. (MK629764) were used as outgroup. All chloroplast genomes were aligned using MAFFT v7.313 (Katoh and Standley, 2013). The ambiguous alignment regions were trimmed using trimAl tool of PhyloSuite (Zhang et al., 2020). The phylogenetic analyses were performed using Maximum likelihoods (ML) and Bayesian inference (BI) methods based on whole chloroplast genomes. The optimal model GTR+F+I+G4 was calculated by Modelfinder. ML analyses were performed by IQ-tree v1.6.8, and the sampling was repeated 1000 times. BI analyses were performed by MrBayes v3.2.8. The result was visualized with FigTree v1.4.4 (http://tree.bio.ed.ac.uk/software/figtree/).

### 2.6 High-performance liquid chromatography analysis

The underground part of samples was washed, dried, ground into powders (50 mesh) and stored under dry conditions at room temperature until analysis. Standard compounds (purity≥98%) of nodakenin, peucedanol, umbelliferone, psoralen, xanthotoxin, bergapten, imperatorin, praeruptorin A, praeruptorin B, praeruptorin E were purchased from Chengdu Lemeitian Pharmaceutical Technology Co., Ltd. (Chengdu, China). Ultrapure water was prepared using a Direct-Pure Water System (RephiLe, Shanghai, China). HPLC-grade acetonitrile was supplied by Oceanpak (Gothenburg, Sweden). Other reagents were analytical grade.

The standard compounds were weighed and dissolved in pure methanol, and make the concentration of each standard solution approximately 1.0 mg/mL. Then, in order to construct the calibration curves, each standard solution was diluted to gradient concentrations with methanol. The powdered sample (0.2 g) was mixed with 10mL methanol, subjected to ultrasonic (40 kHz, 200 W) treatment for 30 min, and allowed to cool, the methanol was added to compensate for the lost weight. The mixture was filtered and the filtrate was collected as the solution to be tested. Then, all solutions were filtered through a 0.45 μm millipore filter and stored at 4°C until analysis (Chu et al., 2020).

An Agilent 1260 Infinity II Diode Array Detector WR (Agilent Technologies Inc., Santa Clara, United States) equipped with an Agilent 5 HC-C_18_ column (4.6 mm × 250 mm, 5 µm) was used for High-performance liquid chromatography (HPLC) analysis. Chromatographic separation was conducted in a flow rate of 1.0 mL/min at 28°C with a mixture of acetonitrile (A) and water (B) as the mobile phase. The gradient elution condition was optimized as follows: 0–10 min, 20%–40% A; 10–12 min, 40%–45% A; 12–17 min, 45%–65% A; 17–40 min, 65% A; 40–43 min, 65%–20% A; 43–50 min, 20% A. The injection volume was 20 μL. The detection wavelength was 297 nm for 1–23 min, and 321 nm for 23–50 min (Chen et al., 2019; Chu et al., 2020). TBtools software (Chen et al., 2020) was used to generate a heatmap based on a hierarchical clustering analysis.

## 3 Results

### 3.1 Textual research on illustrations of Qianhu in the Materia Medica

*Qianhu* was first recorded by *Ming Yi Bie Lu* (approximately 500 A.D.), thus having a medicinal history of more than 1500 years. We reviewed the records of *Qianhu* in the Materia Medica, focusing on its medicinal plants. There are many narrative descriptions of *Qianhu*, but only a few illustrations of *Qianhu* are present. The method of combining pictures and text provides more accurate evidence for exploring the origin of *Qianhu*.

*Ben Cao Tu Jing* (1061) of the Song Dynasty described the morphology of the original *Qianhu* plant, documenting five illustrations from different locations (**Supplementary Figure 2**). The roots of *Zizhou Qianhu* are conical and branched, with a multistrip erect stem, alternate and bipinnate compound leaves, and small leaves. During the Song Dynasty, the jurisdiction of Zizhou included Zichuan, Zouping, Gaoqing County, and Zibo City in Shandong Province, where three species of *Peucedanum* are found. The original *Zizhou Qianhu* plants were similar to *P. wawrae*. *Jianghzou Qianhu* has a stout conical root with residual petiole fibers, solitary stem branches, alternate leaves, bipinnate compound leaves, and compound umbellate inflorescence. The areas under Jiangzhou’s jurisdiction during the Song Dynasty included Xinjiang, Quwo, Xiangfen, Yicheng, Jishan, Jiangxian, and Yuanqu counties in Shanxi Province, where *P. harry-smithii* is distributed and has morphological characteristics consistent with that of “*Jiangzhou Qianhu*.” *Jiangzhou Qianhu* has conical and branched roots, a blade that is oblong-ovate to broadly triangular-ovate, 2–3-ternate-pinnate leaves, and compound umbellate inflorescences. This plant is not a *Peucedanum* but should belong to the Apiaceae family, specifically *Ostericum citriodorum* (Hance) Yuan et Shan. *Qianhu* in Chengzhou has a thick main root, an erect stem, a stem base with residual petiole fibers, alternate and broad leaves, three compound leaves, and a compound umbellate inflorescence terminal. Undoubtedly, it belongs to Apiaceae, but it is not a plant of the genus *Peucedanum*, according to its leaf shape. The root of *Qianhu* in the Jiangning Prefecture is conical, with a striped single leaf without a petiole, and a stem base covered with petiole residual fibers. The morphology is similar to that of Chaihu in Jiangning Prefecture, so the picture should be of the *Bupleurum* L. plant.

The root of *Qianhu* recorded by *Jiu Huang Ben Cao* (1406) of the Ming Dynasty is multibranched, with a single erect stem, alternate leaves, bipinnate compound leaves, parted leaves, and a compound umbellate inflorescence. Based on these morphological characteristics, it is speculated that the original plant may be *P. praeruptorum*. The picture of “*Zizhou Qianhu*” by *Ben Cao Pin Hui Jing Yao* (1505) is similar to the picture of “*Zizhou Qianhu*” by *Ben Cao Tu Jing*. Thus, we speculated that this species may be *P. wawrae* based on its morphological characteristics.

**Figure 1:**
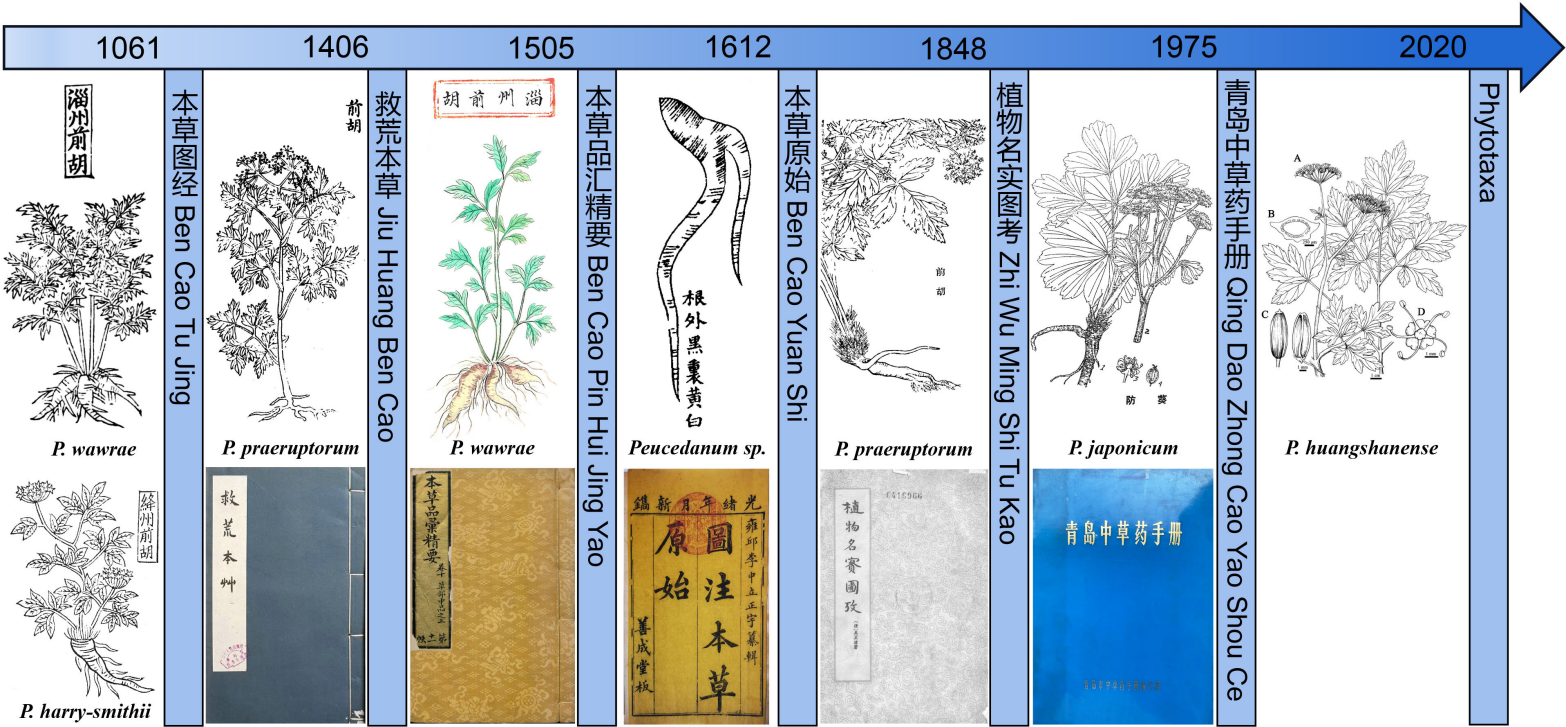
The medicinal plants illustrations of Qianhu in the Materia Medica.

*Ben Cao Yuan Shi* (1612) drew a diagram of the medicinal herbs of *P. praeruptorum*. The root is conical with branches, and the end gradually tapers. It is marked with the words “the root is black outside, yellow or white inside,” which is similar to the description of *Qianhu* by *Jiu Huang Ben Cao*, so it is speculated that the plant is *P. praeruptorum*. In the diagram of “*Qianhu*” painted by *Zhi Wu Ming Shi Tu Kao* (1848) of the Qing Dynasty, the root is thick and branching, and the end tapers gradually. It has several erect stems, small leaves parted deeply, and a compound umbellate inflorescence, and there is the word “*Qianhu*” in the painting; thus, it is speculated to be *P. praeruptorum*. The original plant of “*Fangkui*” by *Qing Dao Zhong Cao Yao Shou Ce* is *P. japonicum* (**Figure 1**). Therefore, it can be inferred from the literature that at least four species of *Peucedanum* have been recorded for medicinal applications.

### 3.2 Phylogenetic analysis based on the ITS sequence

In the present study, we obtained ITS fragments from 18 accessions of the genus *Peucedanum*. The genetic distance among 17 species of *Peucedanum* ranged from 0–0.2578 (**Supplementary Table 2**). The genetic distance between *P. huangshanense* and other species was large, ranging from 0.0714–0.2578. The genetic distance between *P. huangshanense* and *P. insolens* was the greatest, with a value of 0.2578. The genetic distance between *P. huangshanense* and *P. elegans* was 0.2302. In addition, the genetic distances between *P. huangshanense* and *P. longshengense*, as well as *P. huangshanense* and *P. formosanum* Hayata, were the closest, with a value of 0.0714. A total of 44 species from Apiaceae were used for phylogenetic analysis, including *Cicutavirosa* L. (OL473017) and *Cryptotaenia japonica* Hassk. (MH711328), which were used as the outgroup. The alignment matrix contained 732 positions and ranged in length from 698 bp to 725 bp. Phylogenetic analysis (**Supplementary Figure 3**) revealed that the genus *Peucedanum* was not a monophyletic group because its accessions were mixed with other species. However, the samples of *P. huangshanense* were clustered into a monophyletic group by approximately 100% bootstrap values, and they had the closest relationship with *P. longshengense* and *P. formosanum*.

### 3.3 Assembly, annotation, and feature analysis of chloroplast genomes

In this study, we assembled six chloroplast genomes from *P. huangshanense*, *P. praeruptorum*, *P. japonicum*, *P. medicum*, *P. wawrae*, and *P. harry-smithii*. Seven other chloroplast genomes of the genus *Peucedanum* were downloaded from NCBI, including *P. longshengense* (OK336479), *P. harry-smithii* var. *grande* (K. T. Fu) Shan et Sheh (OK336476), *P. delavayi* Franch. (MT843765), *P. angelicoides* Wolff ex Kretschm. (OK336474), *P. ampliatum* K. T. Fu (OK336475), *P. mashanense* Shan et al. (OK336478), and *P. terebinthaceum* (MT671397).

**Figure 2:**
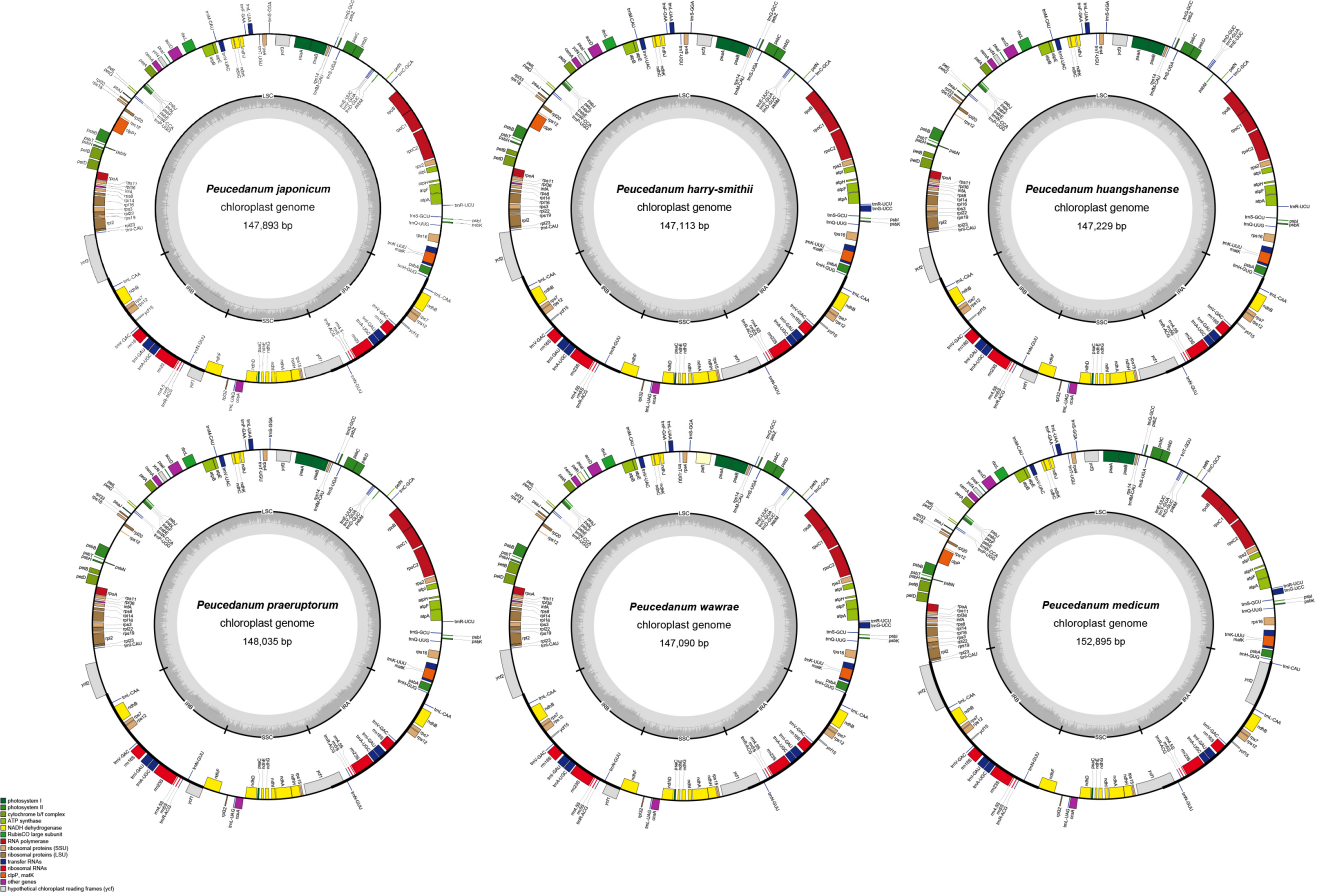
Chloroplast genome maps of *Peucedanum*. Genes shown outside of the outer layer circle are transcribed clockwise, while those insides are transcribed counterclockwise. The genes belonging to different functional groups are color-coded. The dark gray area of the inner circle denotes the GC content of chloroplast.

These 13chloroplast genomes (142,494–155,552 bp, 37.4–37.7% GC) have a typical quadripartite structure consisting of a large single-copy region (LSC, 85,276–99,934 bp), a small single-copy region (SSC, 17,372–17,658 bp), and a pair of inverted repeat regions (IRa and IRb; 12,594–25,394 bp) (**Figure 2**). They contained 114 genes, including 80 protein-coding genes, four rRNA genes, and 30 tRNA genes (**Table 1**; **Supplementary Table 3**). However, *rps19* was missing from *P. delavayi*, and *trnT*-GGU was missing from *P. praeruptorum* and *P. harry-smithii*.

**Table 1:** Comparison of chloroplast features among *Peucedaum* plants.

Taxon	Total length(bp)	LSC(bp)	SSC(bp)	IR (bp)	Total GC content (%)	Total genes (unique)	Protein coding genes (unique)	rRNA genes (unique)	tRNA genes (unique)
*P. praeruptorum*	148,035	93,149	17,630	18,628	37.5	113	80	4	29
*P. harry-smithii*	147,113	91,989	17,560	18,809	37.6	114	80	4	30
*P. huangshanense*	147,229	92,513	17,554	18,581	37.6	114	80	4	30
*P. medicum*	152,895	86,705	17,658	24,266	37.5	114	80	4	30
*P. wawrae*	147,090	92,136	17,528	18,713	37.6	114	80	4	30
*P. japonicum*	147,893	93,287	17,458	18,574	37.5	114	80	4	30
P. delavayi*	155,552	85,276	17,394	16,441	37.6	113	79	4	29
P. angelicoides*	142,494	99,934	17,372	12,594	37.4	114	80	4	30
P. ampliatum*	147,403	92,526	17,519	18,679	37.6	114	80	4	30
P. mashanense*	154,230	86,957	17,572	25,394	37.4	114	80	4	30
P. longshengense*	147,967	93,265	17,576	18,565	37.5	114	80	4	30
*P. harry-smithii* var. grande*	147,046	92,135	17,627	18,642	37.6	113	79	4	29
P. terebinthaceum*	147,925	93,368	17,571	18,493	37.5	114	80	4	30

*Chloroplast genomes were obtained from NCBI.

### 3.4 Chloroplast genome comparison analysis

**Figure 3:**
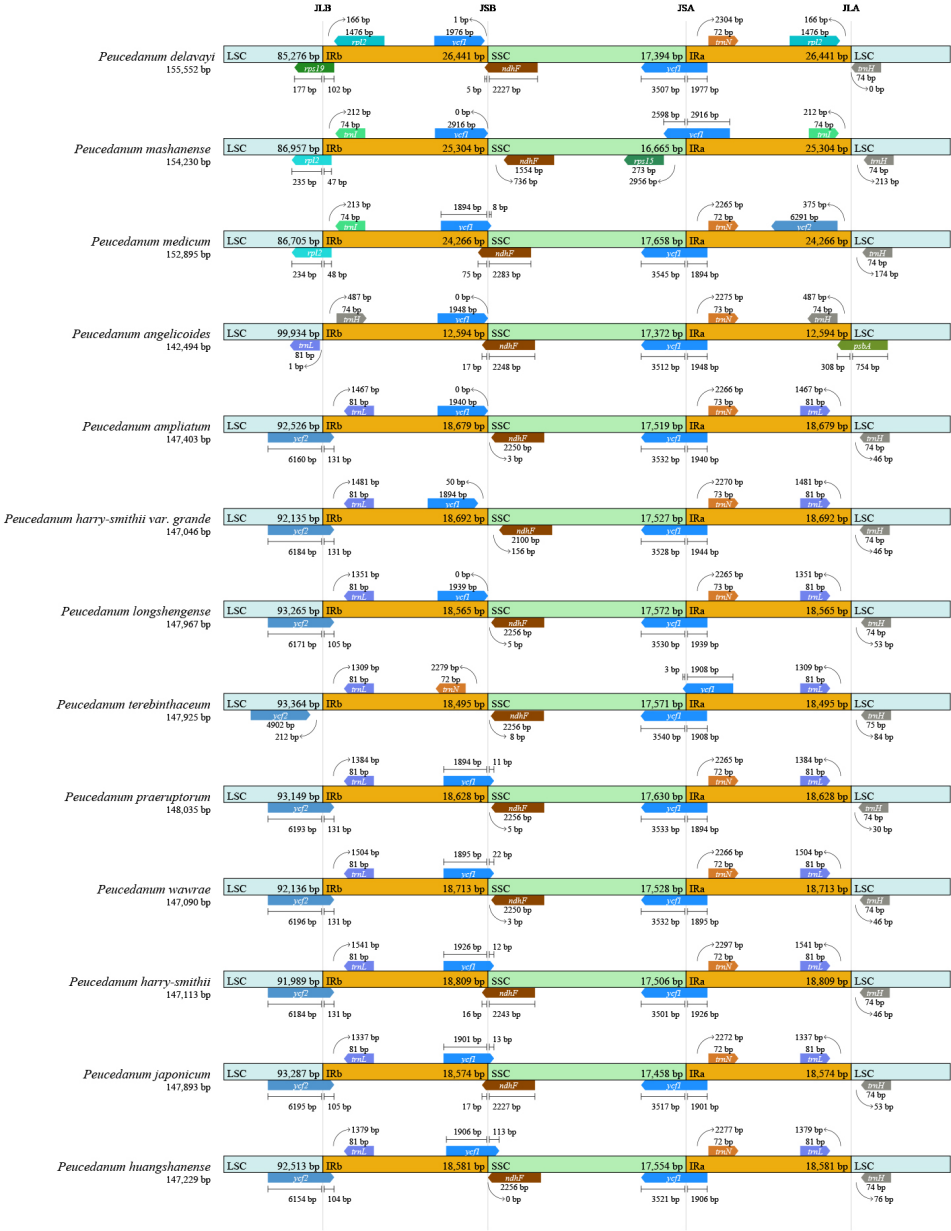
Comparison of the borders of the LSC, SSC, and IR regions among thirteen *Peucedanum* chloroplast genomes.

The borders of IRa/SSC, IRb/SSC, and IRb/LSC among the 13 chloroplast genomes were slightly conserved, and the boundaries of IRb/SSC fell within the *ycf1* gene. The IRa/SSC junctions of most samples were located between the *ycf1* gene and the *ndhF* gene but expanded into the *ndhF* gene in *P. delavayi*, *P. angelicoides*, *P. medicum*, and *P. harry-smithii*. The IRa/LSC borders of most samples were located between genes *trnL* and *trnH*, but they expanded into *psbA* in *P. angelicoides*. However, the junctions of IRb/LSC in chloroplasts within the genus *Peucedanum* were divergent and could be classified into four types. The junctions of IRb/LSC in most remaining plants of *Peucedanum* fell into the *ycf2* gene but contracted to the intergenic regions of *ycf2*-*trnL* in *P. terebinthaceum*, belonging to type I. The IRb/LSC borders fell within the *rps19* gene in *P. delavayi*, which belongs to type II. IRb/LSC borders contracted to the intergenic region of *trnV*-*trnH* in *P. angelicoides* (type III) and moved to the intergenic regions of *rpl23* in *P. mashanense* and *P. medicum* (type IV) (**Figure 3**).

The total number of Simple Sequences Repeat (SSR) ranged from 57 to 89 in 13 *Peucedanum* chloroplast genomes. Most of the SSRs were distributed in the LSC region for all chloroplasts (**Supplementary Figure 4A**). Among these SSRs, mononucleotide repeats were the most abundant (28–54), followed by dinucleotides (14–20) (**Supplementary Figure 4B**). In addition, bases A and T were the dominant elements for all SSRs identified in the 13 chloroplast genomes, based on the MIAS analysis results.

**Figure 4:**
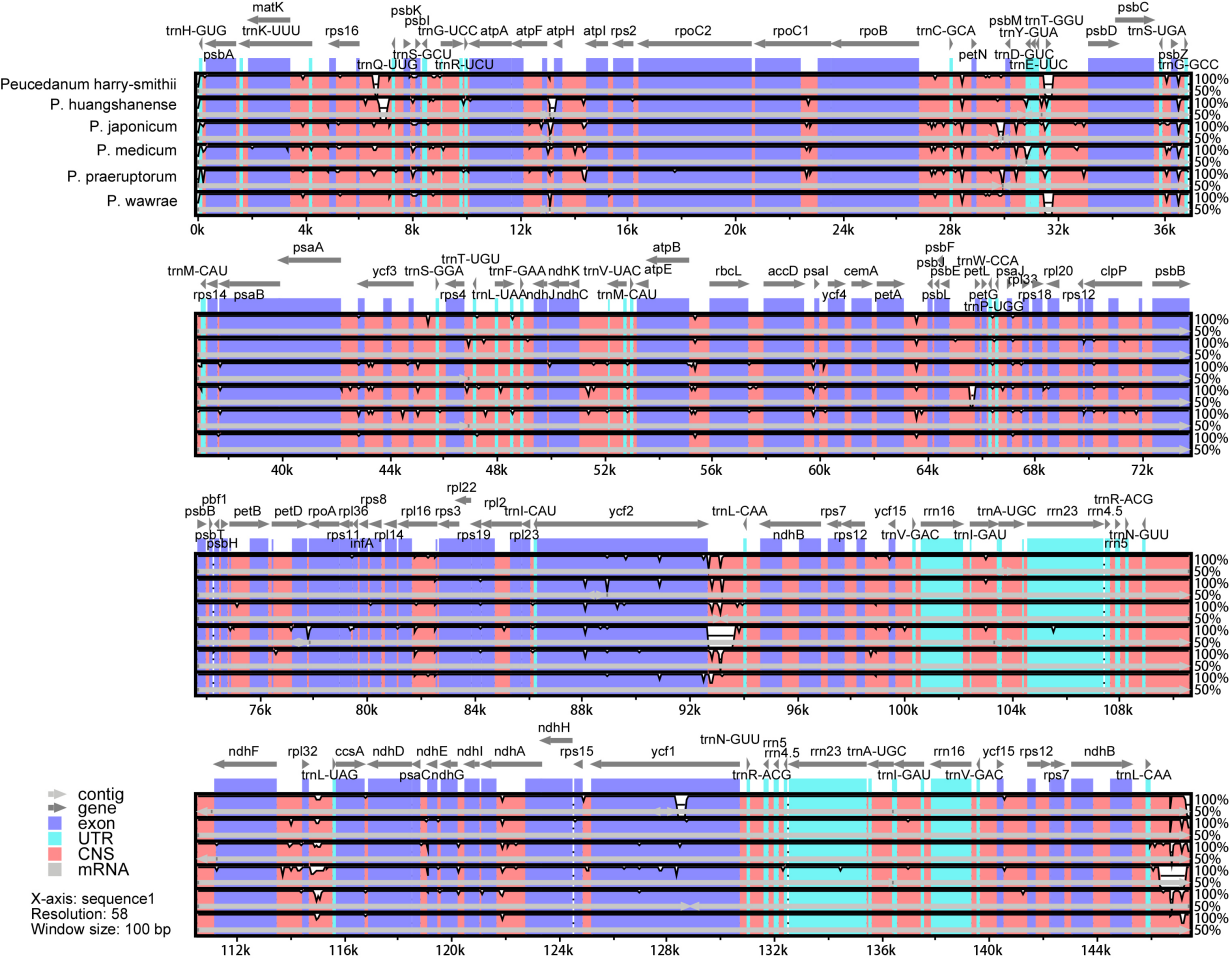
Sequence alignment of the whole chloroplast genomes of six taxa of *Peucedanum* using the LAGAN alignment algorithm in mVISTA, with *P. ampliatum* as the reference. Grey arrows and thick black lines above the alignment indicate gene orientation; The Y-axis represents percent identity in the 50—100% range; Purple bars represent exons, blue bars represent UTRs, and pink bars represent non-coding sequences.

According to the results of the collinearity analysis, the gene arrangement of the 13 *Peucedanum* chloroplast genomes was relatively conserved (**Supplementary Figure 5**). The sequence identities of the six chloroplast genomes assembled in this study were compared using mVISTA, with *P. angelicoides* (OK336474) as a reference. The results indicated that the chloroplast genomes of all *Peucedanum* species showed a high degree of conservation (**Figure 4**). According to the sequence divergences, the 14 mutation hotspot regions were selected as candidate DNA barcodes, including nine protein-coding genes (*matK*, *rps16*, *petL*, *psbH*, *rps8*, *rpl22*, *ycf2*, *ycf1*, and *rpl32*; all with Pi > 0.00800) (**Supplementary Figure 6A**) and five internal gene space genes (*psbA*-*trnK*, *trnH*-*psbA*, *ycf2*-*trnL*, *psbK*-*psbI*, and *ycf4*-*cemA*; all with Pi > 0.03000) (**Supplementary Figure 6B**).

### 3.5 Phylogenetic analysis based on chloroplast genomes

**Figure 5:**
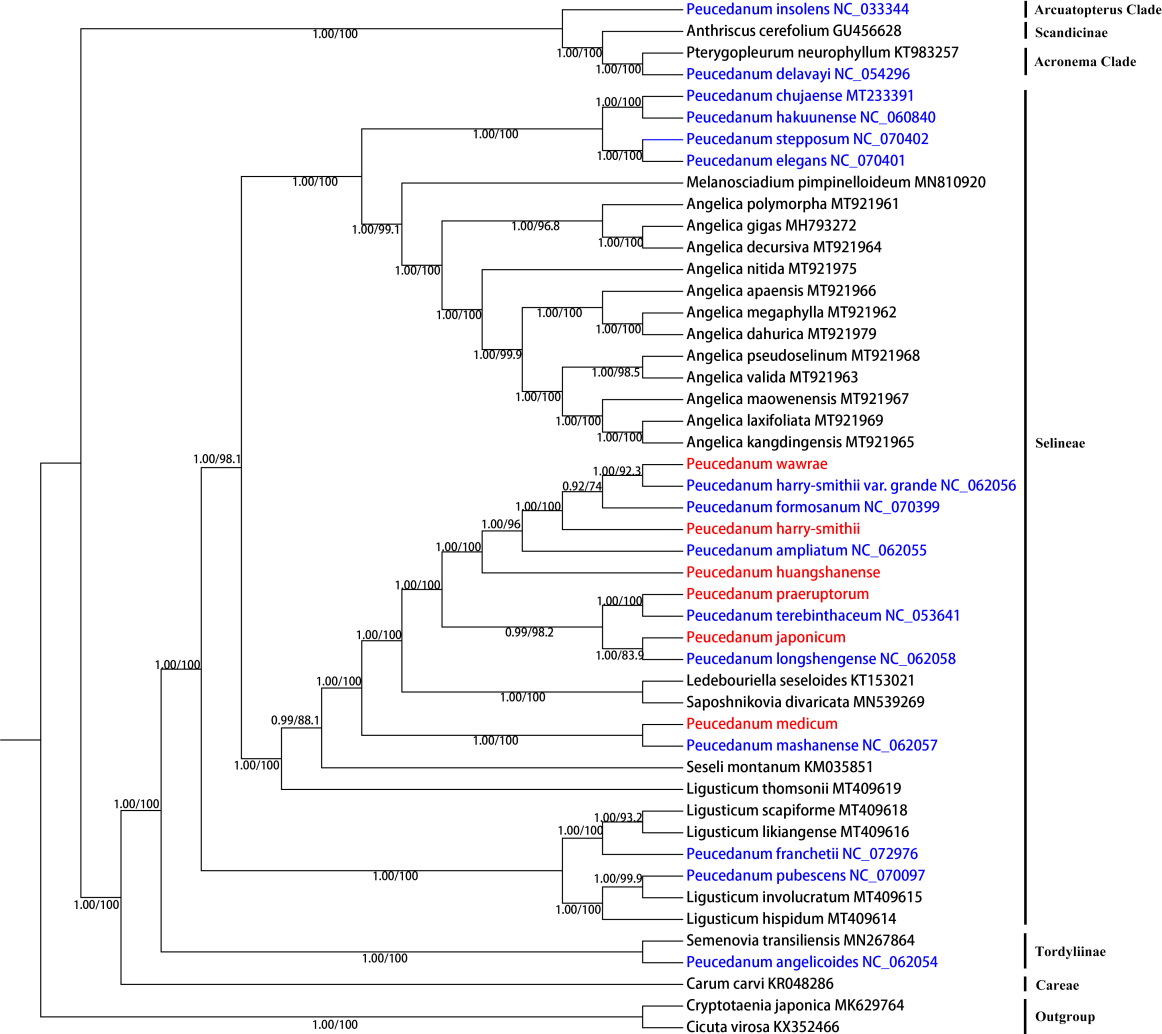
Phylogenetic relationships inferred from maximum likelihood (ML) and Bayesian inference (BI) analyses based on the complete chloroplast genomes. Numbers represent Bayesian posterior probabilities (PP) and maximum likelihood bootstrap values (BS).

In this study, based on complete chloroplast genomes, 48 Apiaceae species were used for phylogenetic analysis. These topologies of the ML and BI trees were consistent (**Figure 5**). Both analyses robustly supported that the accessions of the *Peucedanum* genus were not clustered as monophyletic but fell into four clades. Most of the species of *Peucedanum* belonged to the tribe Selineae, while the samples of Selineae were also not clustered into a clade. *P. franchetii* and *P. pubescens* clustered into a clade with Ligusticum L., which was distant from all other *Peucedanum* members. *P. chujaense*, *P. hakuunense*, *P. elegans*, and *P. stepposum* clustered into a clade. *P. mashanense* was clustered with *P. medicum*, while *P. wawrae*, *P. harry-smithii* var. *grande*, *P. formosanum* (IUCN Red List of Threatened Species, NT), *P. harry-smithii*, *P. ampliatum* (IUCN, CR), *P. huangshanense*, *P. praeruptorum*, *P. terebinthaceum*, *P. japonicum*, and *P. longshengense* also formed a clade. First, the sister groups of *P. praeruptorum* and *P. terebinthaceum*, *P. longshengense* and *P. japonicum*, diverged from the others. Then, *P. huangshanense*, *P. ampliatum*, *P. harry-smithii*, *P. formosanum*, *P. harry-smithii* var. *grande*, and *P. wawrae* were separated. According to the results of the phylogenetic analysis based on the complete chloroplast genome, *P. huangshanense* had the closest genetic relationship to *P. ampliatum*, followed by *P. harry-smithii*. In addition, *P. insolens* belonged to the Arcuatopterus clade. *P. delavayi* was found to be a sister group of *Pterygopleurum neurophyllum* (Maxim.) Kitag., which belonged to the Acronema clade. *P. angelicoides* was a sister group of *Semenovia transiliensis* Regel & Herder belonging to the Tordyliinae clade.

### 3.6 Quantitative determination of ten coumarins in the *Peucedanum* species

**Figure 6:**
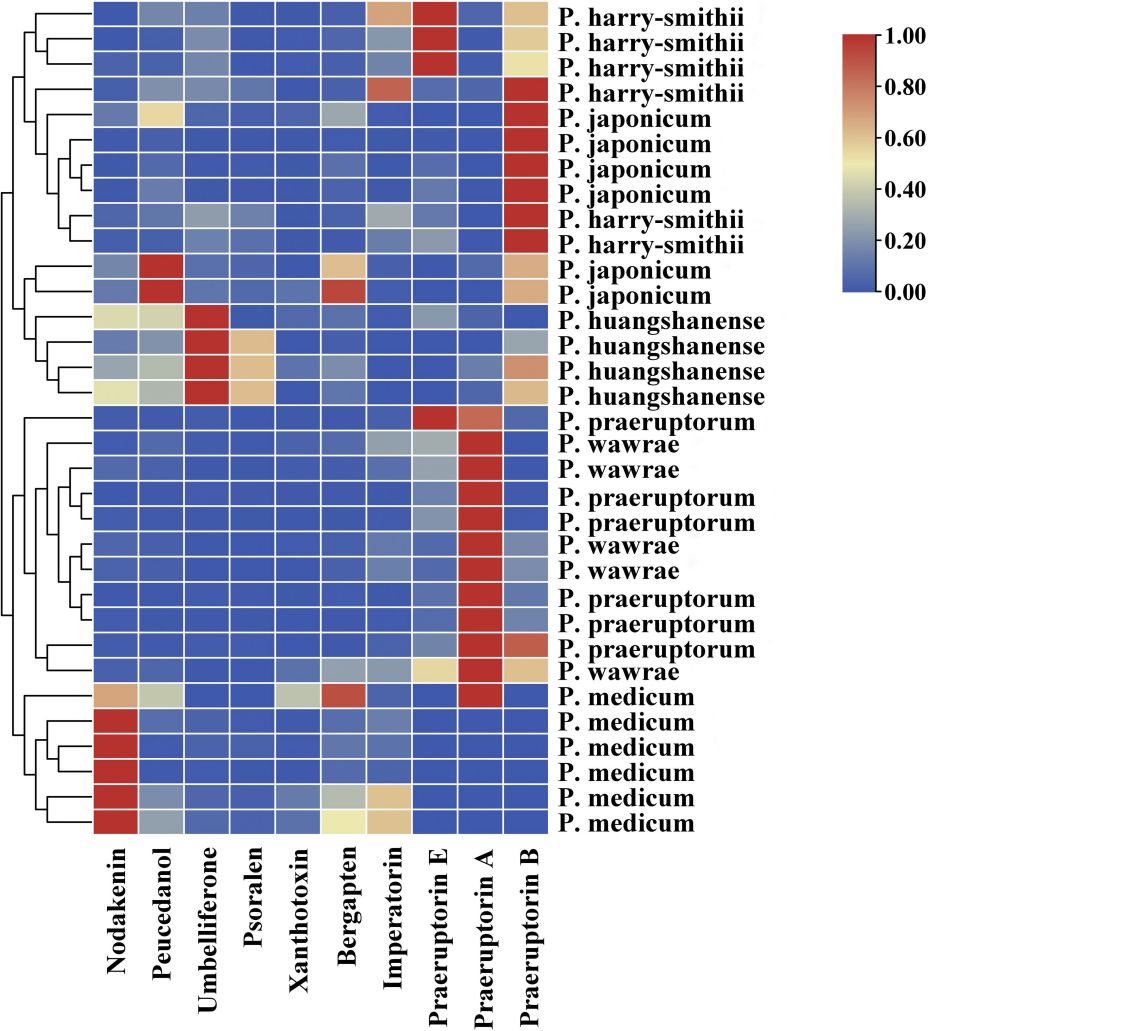
Clustering heatmap of ten compounds in six *Peucedanum* species.

A further quantitative analysis was performed to explore the differences in the components between the underground parts of the plants of the *Peucedanum* genus. We simultaneously determined the ten components of the samples using HPLC, and the results are shown in **Figure 6** and **Supplementary Table 4**. The contents of the ten compounds were different in these samples. Ten compounds were found in *P. praeruptorum*, *P. wawrae*, and *P. harry-smithii*, but the first three compounds were not detected in some samples. Nodakenin was not detected in a *P. praeruptorum*, whereas xanthotoxin was not detected in another *P. praeruptorum*. Umbelliferone, psoralen, and xanthotoxin were not detected in few *P. wawrae*, and praeruptorin B was not detected in some *P. wawrae*. Three samples of *P. harry-smithii*—nodakenin, psoralen, and xanthotoxin—were not detected separately. Praeruptorin E was not detected in the samples of *P. japonicum.* Xanthotoxin was not detected in one *P. japonicum*, but praeruptorin A was detected in this sample. None of the *P. medicum* isolates contained praeruptorin B or E, and praeruptorin A was detected only in few *P. medicum*, which lacked umbelliferone and Psoralen.

The praeruptorin A content in most of *P. praeruptorum* was the highest. The praeruptorin E content in a small amount of *P. praeruptorum* was the highest, followed by praeruptorin A. The content of praeruptorin B was the second highest in some *P. praeruptorum*, but the content of praeruptorin E was the second highest in other *P. praeruptorum*. In individual *P. japonicum* the peucedanol content was the highest, followed by praeruptorin B. The contents of these two components were the opposite in other *P. japonicum*. Nodakenin content was highest in most *P. wawrae*. The content of bergapten or imperatorin was the second highest and praeruptorin B was the highest in half of *P. harry-smithii*, while the content of praeruptorin E was the highest in the other half of *P. harry-smithii*. The umbelliferone content in *P. huangshanense* was the highest, followed by psoralen and praeruptorin B. xanthotoxin, bergapten, and xanthotoxin were present only in small amounts in *P. huangshanense*. According to the cluster results, among the six species of the *Peucedanum* genus, the contents of various compounds in *P. japonicum* and *P. harry-smithii* were relatively close, *P. huangshanense* was similar to them, *P. wawrae* and *P. praeruptorum* were relatively close to each other, and *P. medicum* was a single branch.

## 4 Discussion

### 4.1 Speculation of the medicinal value of *P huangshanense*

*Qianhu* has a long history as a traditional Chinese medicine. Therefore, illustrations of the medicinal plants of *Qianhu* were found in ancient herbal books. According to the results of textual research on illustrations in Bencao literature, it can be inferred that at least five species of *Peucedanum* have been used as the original plants of *Qianhu*. Therefore, we collected these species of *Peucedanum* for our research.

The phylogenetic tree based on ITS sequences showed that the genetic relationship between *P. huangshanense* and the sister group of *P. longshengense* and *P. formosanum* was closest (**Figure 3**). However, the result of the phylogenetic analysis based on the whole chloroplast genome revealed that the genetic relationship between *P. huangshanense* and *P. ampliatum* was closest, followed by the relationship between *P. huangshanense* and *P. harry-smithii* (**Figure 5**). From the results of the cluster analysis of the compound content, the components of *P. huangshanense* were the most similar to those of *P. japonicum* and *P. harry-smithii*. Pharmacologists believe that medicinal plants containing similar chemical components often have similar clinical efficacies (Hao and Xiao, 2020; Gong et al., 2022). *Chinese Herbal Medicine* records that *P. japonicum* has the effects of clearing heat and relieving cough, diuresis, and detoxification. Yingqianhu has the effects of reducing Qi, resolving phlegm, dispersing wind, and clearing heat. It is often used to treat wind-heat cough, thick sputum, chest tightness, and asthma (Zhang, 2020). The Standard of Traditional Chinese Medicine in Shaanxi Province (2015 edition) states the plant sources of Yingqianhu include *P. harry-smithii*, *P. harry-smithii* var. *subglabrum*, and *P. harry-smithii* var. *grande* (Qiu et al., 2019). Therefore, we think that *P. harry-smithii* has the same effect as Yingqianhu. Based on the efficacy of *P. japonicum* and *P. harry-smithii*, we speculated that *P. huangshanense* could evacuate wind heat, relieve cough, and resolve phlegm.

According to the analysis of 10 coumarins in the six *Peucedanum* species, the content of umbelliferone in *P. huangshanense* was highest, followed by psoralen and praeruptorin B. Umbelliferolide (7-hydroxycoumarin) has various pharmacological properties, such as antioxidation, anti-hyperglycemia, antitumor, anti-inflammatory, anti-hyperlipidemia, immunomodulatory, hemostatic, and antiulcerogenic properties (Li et al., 2018; Cruz et al., 2020). Psoralen has been reported to be used to inhibit tumors, for example central nervous system malignant tumors, breast cancer, erythroleukemia, and oral carcinoma, as well as having anti-inflammatory, anti-diabetic, anti-microbial, and osteogenesis effects (Thakur et al., 2020). Praeruptorin B can inhibit osteoclast formation, thus treating osteoporosis and exerting anti-cancer effects (Lin et al., 2020; Xu et al., 2022). Pyranocoumarin can relieve neuropathic pain and neuroinflammatory reactions and exert anti-inflammatory effects (Onder and Trendafilova, 2022). Based on the main coumarin components of *P. huangshanense*, it can be speculated that *P. huangshanense* may has effects of anti-cancer, anti-inflammatory, anti-diabetic, bacteriostatic, and osteoporosis. Furthermore, these effects could be transformed into resolving phlegm to stop cough and dispersing wind heat. However, these specific effects need to be verified through further pharmacological experiments.

### 4.2 Discovery of molecular markers for phylogenetic classification of *Peucedanum*

DNA barcoding is a method used to identify species based on short and standardized DNA fragments (Gao et al., 2019). In most angiosperms, the chloroplast genome is maternally inherited, with a low recombination rate and abundant mutation sites that can accurately identify medicinal plants (Teske et al., 2020). Plastid markers, such as *rbcL*, *matK*, *psbA*, and *trnH*, have been widely used in medicinal species identification (Mahima et al., 2022). At present, 12 chloroplast genomes of the genus *Peucedanum* have been compared to screen hotspot mutation regions, providing potential molecular markers for species division and population genetic research of *Peucedanum* (Liu et al., 2022).

In this study, six complete chloroplast genomes of the genus *Peucedanum* were assembled, and the complete chloroplast genomes of *P. huangshanense* and *P. harry*-*smithii* were published for the first time. Comparison of 13 chloroplast genomes of the genus *Peucedanum* showed relatively conservative genome structures, and all had typical tetrad structures. In terms of gene number—except for one gene deletion in *P. delavayi*, *P. praeruptorum*, and *P. harry-smithii* var. *grande*—the gene number of other species were consistent. The distribution of Simple Sequences Repeat (SSR) was also consistent, with the LSC region being the most distributed, and the IR and SSC regions being less distributed. In addition, we observed obvious diversity in the 13 chloroplast genomes of *Peucedanum*. First, the chloroplast genome size varied greatly from 142,494 bp (*P. angelicoides*) to 155,552 bp (*P. delavayi*). Second, it can be seen from the map of *Peucedanum* chloroplast genomes that there are two *ycf2* genes in *P. medicum*, and there is an inversion of the *trnD-trnY-trnE* gene in *P. huangshanense*, which can be used as a specific molecular marker for species identification. Third, SSR analysis showed that only a small number of hexanucleotide repeats were found in *P. japonicum*, *P. praeruptorum*, *P. longshengense*, and *P. mashanense*. The number of trinucleotide repeats in *P. angelicoides* was 10 and only 2–4 in the other species. In addition, we found 14 hotspot mutation regions in the sliding window analysis, which has the potential to develop DNA barcoding for the identification of *Peucedanum* species.

During the evolution of the chloroplast genome, the differences of SC/IR boundaries between different species are often observed, which further leads to change in the size of the chloroplast genomes. In this study, the IR regions of *P. delavayi*, *P. mashanense*, and *P. medicum* expanded the most, while the complete chloroplast genomes became larger, and the LSC region also changed greatly. The regions of IR are the most conserved parts of chloroplast genomes, but their contraction and expansion can account for the size differences between them. Therefore, IR regions can be used to explain variations in chloroplast genome size (Liu et al., 2019; Xue et al., 2019). In this study, 13 species of *Peucedanum* were roughly divided into four types according to the IR boundary analysis results, which also provided a basis for the species classification of *Peucedanum*.

In addition, based on both ITS and the chloroplast genomes, the phylogenetic trees showed that the genus *Peucedanum* is not monophyletic but divided into four clades. Most species of *Peucedanum* are distributed in the Selenae Clade, while the samples of Selineae were also not clustered into a clade. This conclusion supports the results of previous research (Liu et al., 2022). The phylogenetic tree based on ITS and chloroplast genome was slightly different. And the result of the phylogenetic analysis is also different from the morphological classification of *Peucedanum*, which indicates that the systematic classification of *Peucedanum* requires further study.

## 5 Conclusion

In this study, on the basis of textual research on the illustration of *Qianhu* in Bencao literature, we chose five medicinal of *Peucedanum* and *P. huangshanense* together for subsequent research. Six complete chloroplast genomes were successfully assembled, and chloroplast genomes of *P. huangshanense* and *P. harry-smithii* were reported for the first time. Phylogenetic analysis showed that the phylogenetic tree based on the complete chloroplast genome had high support and resolution, and the genetic relationship between *P. huangshanense* and *P. ampliatum* was closest, followed by the relationship between *P. huangshanense* and *P. harry-smithii*. In addition, ten coumarins from the samples were analyzed using HPLC. The results of chemical composition clustering showed that the contents and types of compounds in *P. huangshanense*, *P. japonicum*, and *P. harry-smithii* were the most similar. Overall, our research has laid the foundation for future studies on the utilization of *P. huangshanense* in traditional medicine. This can also be used as a reference for the research of new medicinal resources.

## Data availability statement

The datasets presented in this study can be found in online repositories. The names of the repository/repositories and accession number(s) can be found in the article/**Supplementary Material.**

## Author contributions

HS, LH, and HP conceived and designed the experiments. HS, SC, and LJ performed the experiments and writing—original draft. HS and ZT validated and analyzed the data. HP investigated and collected the samples. M’eC guided the literature research. All authors contributed to the article and approved the submitted version.
