# Supplementary material for: Integrative analysis of chloroplast genome, chemicals, and illustrations in Bencao literature provides insights into the medicinal value of Peucedanum huangshanense
Source: Front Plant Sci. 2023 Aug 4;14:1179915. doi: 10.3389/fpls.2023.1179915 (PMC10436485; doi:10.3389/fpls.2023.1179915)
Supplement: Supplementary file 1 [file DataSheet_1.docx]

**Supplementary Table 1 |** Collection information and GenBank accession number of samples.

| No. | Taxa | Collection Area | Collection Times | Voucher | GenBank accession number | |
| --- | --- | --- | --- | --- | --- | --- |
|  |  |  |  |  | ITS | cp Genome |
| 1 | *Peucedanum praeruptorum**^△^ | Chizhou, Anhui | 2021.11 | QH211101 | OP851693 | OQ473756 |
| 2 | *P. praeruptorum** | Chizhou, Anhui | 2021.11 | QH211102 | OP851694 |  |
| 3 | *P. praeruptorum** | Chizhou, Anhui | 2021.11 | QH211103 | OP851695 |  |
| 4 | *P. praeruptorum* | Chizhou, Anhui | 2021.11 | QH211104 |  |  |
| 5 | *P. praeruptorum* | Chizhou, Anhui | 2021.11 | QH211105 |  |  |
| 6 | *P. praeruptorum* | Chizhou, Anhui | 2021.11 | QH211106 |  |  |
| 7 | *P. huangshanense** | Huangshan, Anhui | 2021.07 | HS210701 | OP851696 |  |
| 8 | *P. huangshanense** | Huangshan, Anhui | 2021.07 | HS210702 | OP851697 |  |
| 9 | *P. huangshanense**^△^ | Huangshan, Anhui | 2021.07 | HS210703 | OP851698 | OQ473753 |
| 10 | *P. huangshanense* | Huangshan, Anhui | 2021.07 | HS210704 |  |  |
| 11 | *P. japonicum** | Fuzhou, Fujian | 2021.10 | BH211001 | OP851699 |  |
| 12 | *P. japonicum** | Fuzhou, Fujian | 2021.10 | BH211002 | OP851700 |  |
| 13 | *P. japonicum**^△^ | Fuzhou, Fujian | 2021.10 | BH211003 | OP851701 | OQ473754 |
| 14 | *P. japonicum* | Fuzhou, Fujian | 2021.10 | BH211004 |  |  |
| 15 | *P. japonicum* | Fuzhou, Fujian | 2021.10 | BH211005 |  |  |
| 16 | *P. japonicum* | Fuzhou, Fujian | 2021.10 | BH211006 |  |  |
| 17 | *P. medicum**^△^ | Yiyang, Hunan | 2021.11 | HZ211101 | OP851702 | OQ473755 |
| 18 | *P. medicum** | Yiyang, Hunan | 2021.11 | HZ211102 | OP851703 |  |
| 19 | *P. medicum** | Yiyang, Hunan | 2021.11 | HZ211103 | OP851704 |  |
| 20 | *P. medicum* | Yiyang, Hunan | 2021.11 | HZ211104 |  |  |
| 21 | *P. medicum* | Yiyang, Hunan | 2021.11 | HZ211105 |  |  |
| 22 | *P. medicum* | Yiyang, Hunan | 2021.11 | HZ211106 |  |  |
| 23 | *P. wawrae** | Chuzhou, Anhui | 2021.11 | TS211101 | OP851705 |  |
| 24 | *P. wawrae**^△^ | Chuzhou, Anhui | 2021.11 | TS211102 | OP851706 | OQ473757 |
| 25 | *P. wawrae** | Chuzhou, Anhui | 2021.11 | TS211103 | OP851707 |  |
| 26 | *P. wawrae* | Chuzhou, Anhui | 2021.11 | TS211104 |  |  |
| 27 | *P. wawrae* | Chuzhou, Anhui | 2021.11 | TS211105 |  |  |
| 28 | *P. harry-smithii**^△^ | Jincheng, Shanxi | 2018.08 | HB180801 | OP851708 | OQ473752 |
| 29 | *P. harry-smithii** | Jincheng, Shanxi | 2018.08 | HB180802 | OP851709 |  |
| 30 | *P. harry-smithii** | Jincheng, Shanxi | 2018.08 | HB180803 | OP851710 |  |
| 31 | *P. harry-smithii* | Jincheng, Shanxi | 2018.08 | HB180804 |  |  |
| 32 | *P. harry-smithii* | Jincheng, Shanxi | 2018.08 | HB180805 |  |  |
| 33 | *P. harry-smithii* | Jincheng, Shanxi | 2018.08 | HB180806 |  |  |

*Samples of DNA extraction, ^△^Samples of chloroplast genome sequencing.

**Supplementary Table 2 |** ITS genetic distance analysis of 17 *Peucedanum* species.

| 样品 | *P.praeruptorum* | *P.huangshanense* | *P.japonicum* | *P.wawrae* | *P.medicum* | *P. harry-smithii* | *P.harry-smithii var. grande* | *P.ampliatum* | *P.delavayi* | *P.insolens* | *P.longshengense* | *P.terebinthaceum* | *P.hakuunense* | *P.formosanum* | *P.elegans* | *P.stepposa* | | *P.mashanense* |
| --- | --- | --- | --- | --- | --- | --- | --- | --- | --- | --- | --- | --- | --- | --- | --- | --- | --- | --- |
| *P.praeruptorum* |  |  |  |  |  |  |  |  |  |  |  |  |  |  |  | |  |  |
| *P.huangshanense* | 0.1003 |  |  |  |  |  |  |  |  |  |  |  |  |  |  | |  |  |
| *P.japonicum* | 0.0271 | 0.0962 |  |  |  |  |  |  |  |  |  |  |  |  |  | |  |  |
| *P.wawrae* | 0.0017 | 0.0984 | 0.0254 |  |  |  |  |  |  |  |  |  |  |  |  | |  |  |
| *P.medicum* | 0.0237 | 0.0884 | 0.0134 | 0.0220 |  |  |  |  |  |  |  |  |  |  |  | |  |  |
| *P.harry-smithii* | 0.0000 | 0.1003 | 0.0271 | 0.0017 | 0.0237 |  |  |  |  |  |  |  |  |  |  | |  |  |
| *P.harry-smithii var. grande* | 0.0017 | 0.1002 | 0.0288 | 0.0033 | 0.0254 | 0.0017 |  |  |  |  |  |  |  |  |  | |  |  |
| *P.ampliatum* | 0.0000 | 0.0950 | 0.0271 | 0.0017 | 0.0237 | 0.0000 | 0.0016 |  |  |  |  |  |  |  |  | |  |  |
| *P.delavayi* | 0.0396 | 0.0967 | 0.0325 | 0.0379 | 0.0255 | 0.0396 | 0.0414 | 0.0396 |  |  |  |  |  |  |  | |  |  |
| *P.insolens* | 0.1956 | 0.2578 | 0.1912 | 0.1935 | 0.1800 | 0.1956 | 0.1973 | 0.1952 | 0.1987 |  |  |  |  |  |  | |  |  |
| *P.longshengense* | 0.0394 | 0.0714 | 0.0359 | 0.0377 | 0.0289 | 0.0394 | 0.0412 | 0.0394 | 0.0360 | 0.1932 |  |  |  |  |  | |  |  |
| *P.terebinthaceum* | 0.0325 | 0.0762 | 0.0220 | 0.0308 | 0.0152 | 0.0325 | 0.0334 | 0.0305 | 0.0204 | 0.1803 | 0.0203 |  |  |  |  | |  |  |
| *P.hakuunense* | 0.0360 | 0.0846 | 0.0255 | 0.0343 | 0.0186 | 0.0360 | 0.0378 | 0.0360 | 0.0238 | 0.1825 | 0.0237 | 0.0101 |  |  |  | |  |  |
| *P.formosanum* | 0.0394 | 0.0714 | 0.0359 | 0.0377 | 0.0289 | 0.0394 | 0.0412 | 0.0394 | 0.0360 | 0.1932 | 0.0000 | 0.0203 | 0.0237 |  |  | |  |  |
| *P.elegans* | 0.1729 | 0.2302 | 0.1753 | 0.1708 | 0.1642 | 0.1729 | 0.1749 | 0.1729 | 0.1732 | 0.2137 | 0.1636 | 0.1557 | 0.1664 | 0.1636 |  | |  |  |
| *P.stepposa* | 0.0521 | 0.1041 | 0.0413 | 0.0503 | 0.0325 | 0.0521 | 0.0527 | 0.0509 | 0.0432 | 0.1979 | 0.0466 | 0.0318 | 0.0360 | 0.0466 | 0.1689 | |  |  |
| *P.mashanense* | 0.0289 | 0.0925 | 0.0185 | 0.0272 | 0.0084 | 0.0289 | 0.0301 | 0.0284 | 0.0272 | 0.1862 | 0.0341 | 0.0200 | 0.0237 | 0.0341 | 0.1706 | | 0.0388 |  |

**Supplementary Table 3 |** The chloroplast genomes information of six *Peucedaum* species.

| Category for gene | Group of gene | Name of gene |
| --- | --- | --- |
| Genes for photosynthesis | Subunits of Photosystem I | *psaA*,*psaB*,*psaC*,*psaI*,*psaJ* |
|  | Subunits of Photosystem II | *psbA*,*psbB*,*psbC*,*psbD*,*psbE*,*psbF*,*psbH*,*psbI*,*psbJ*,*psbK*,*psbL*,*psbM*,*psbT*,*psbZ* |
|  | Subunits of NADH oxidoreductase | *ndhA*(2)*,*ndhB*(2)*,*ndhC*,*ndhD*,*ndhE*,*ndhF*,*ndhG,ndhH,ndhI,ndhJ,ndhK* |
|  | Subunits of cytochrome b/f complex | *petA,petB*,petD*,petG,petL,petN* |
|  | Subunits of ATP synthase | *atpA,atpB,atpE,atpF*,atpH,atpI* |
|  | Large subunit of RuBisCo | *rbcL* |
|  | Subunits photochlorophyllide reductase | *-* |
| Self-replication | Large subunit of ribosomal proteins | *rpl14,rpl16*,rpl2*,rpl20,rpl22,rpl23,rpl32,rpl33,rpl36* |
|  | Small subunit of ribosomal proteins | *rps11,rps12**(4),rps14,rps15,rps16*,rps18,rps19,rps2,rps3,rps4,rps7(2),rps8* |
|  | Subunits of RNA polymerase | *rpoA,rpoB,rpoC1*,rpoC2* |
|  | Ribosomal RNA | *rrn16(2),rrn23(4),rrn23-fragment(2),rrn4.5(2),rrn5(2)* |
|  | Transfer RNA | *trnA-UGC*(2),trnC-GCA,trnD-GUC,trnE-UUC,trnF-GAA,trnG-GCC,trnG-UCC*,trnH-GUG,trnI-CAU,trnI-GAU*(2),trnK-UUU*,trnL-CAA(2),trnL-UAA*,trnL-UAG*,*trnM-CAU*,*trnN-GUU(2),trnP-UGG,trnQ-UUG,trnR-ACG(2),trnR-UCU,trnS-GCU,trnS-GGA,trnS-UGA,trnT-GGU,trnT-UGU,trnV-GAC(2),trnV-UAC*,trnW-CCA,trnY-GUA,trnfM-CAU* |
| Other genes | Maturase | *matK* |
|  | Protease | *clpP1*** |
|  | Envelope membrane protein | *cemA* |
|  | Acetyl-CoA carboxylase | *accD* |
|  | c-Type cytochrome synthesis gene | *ccsA* |
|  | Translational initiation factor | *infA* |
|  | other | *pafI**(2),pafII,pbf1* |
| Genes unknown function | Conserved open reading frames | *ycf1(3),ycf2* |

Ntron-containing genes are marked by asterisks (*)

**Supplementary Table 4** | Contents of ten coumarins in underground parts of six *Peucedanum* species.

|  | **Nodakenin** | **Peucedanol** | **Umbelliferone** | **Psoralen** | **Xanthotoxin** | **Bergapten** | **Imperatorin** | **Praeruptorin E** | **Praeruptorin A** | **Praeruptorin B** |
| --- | --- | --- | --- | --- | --- | --- | --- | --- | --- | --- |
| **P. praeruptorum 1** | 0.00000 | 0.11278 | 0.23880 | 0.14617 | 0.12629 | 0.18885 | 0.11371 | 1.51484 | 16.57821 | 1.87543 |
| **P. praeruptorum 2** | 0.29385 | 0.13367 | 0.14637 | 0.08883 | 0.05432 | 0.10750 | 0.24607 | 1.36618 | 17.79990 | 2.74194 |
| **P. praeruptorum 3** | 0.15683 | 0.07407 | 0.15500 | 0.09419 | 0.00000 | 0.04902 | 0.33158 | 1.37910 | 8.92466 | 7.77824 |
| **P. praeruptorum 4** | 0.04003 | 0.17612 | 0.28065 | 0.23637 | 0.15036 | 0.23649 | 0.13561 | 2.92197 | 19.92503 | 0.23136 |
| **P. praeruptorum 5** | 0.47527 | 0.23544 | 0.20250 | 0.07721 | 0.06160 | 0.13772 | 0.28042 | 4.38119 | 21.00423 | 0.28077 |
| **P. praeruptorum 6** | 0.22337 | 0.12535 | 0.20451 | 0.01557 | 0.02969 | 0.07786 | 0.35380 | 12.08453 | 10.17002 | 0.70988 |
| **P. huangshanense 1** | 0.10581 | 0.13622 | 0.39991 | 0.24611 | 0.03780 | 0.07324 | 0.00000 | 0.00000 | 0.05386 | 0.29473 |
| **P. huangshanense 2** | 0.15804 | 0.26381 | 1.28832 | 0.79721 | 0.00000 | 0.03255 | 0.00000 | 0.00000 | 0.00000 | 0.34133 |
| **P. huangshanense 3** | 0.23028 | 0.16211 | 0.48990 | 0.30194 | 0.00000 | 0.05040 | 0.00000 | 0.00000 | 0.02732 | 0.30583 |
| **P. huangshanense 4** | 0.20744 | 0.19623 | 0.46012 | 0.00336 | 0.03033 | 0.04213 | 0.00603 | 0.10310 | 0.02071 | 0.00000 |
| **P. japonicum 1** | 0.05568 | 0.33673 | 0.02894 | 0.01599 | 0.00000 | 0.20627 | 0.00968 | 0.00000 | 0.02079 | 0.22164 |
| **P. japonicum 2** | 0.07065 | 0.31585 | 0.02773 | 0.01524 | 0.02690 | 0.15952 | 0.00603 | 0.00000 | 0.00000 | 0.57772 |
| **P. japonicum 3** | 0.06497 | 0.55182 | 0.05723 | 0.03354 | 0.05226 | 0.51916 | 0.01115 | 0.00000 | 0.00000 | 0.36530 |
| **P. japonicum 4** | 0.06895 | 0.62519 | 0.09496 | 0.14232 | 0.07056 | 0.79679 | 0.00603 | 0.70501 | 0.00000 | 9.45928 |
| **P. japonicum 5** | 0.03419 | 0.63030 | 0.03479 | 0.03080 | 0.02233 | 0.20189 | 0.00603 | 0.57097 | 0.00000 | 4.88264 |
| **P. japonicum 6** | 0.05762 | 0.19337 | 0.01180 | 0.02117 | 0.02067 | 0.09980 | 0.00603 | 0.06459 | 0.00000 | 7.41882 |
| **P. medicum 1** | 0.09017 | 0.05068 | 0.00000 | 0.00000 | 0.04845 | 0.12098 | 0.00603 | 0.00000 | 0.13204 | 0.00000 |
| **P. medicum 2** | 0.74631 | 0.14057 | 0.03962 | 0.02261 | 0.09309 | 0.25735 | 0.45242 | 0.00000 | 0.00000 | 0.00000 |
| **P. medicum 3** | 1.08275 | 0.26801 | 0.06873 | 0.04067 | 0.09760 | 0.54085 | 0.65621 | 0.00000 | 0.00000 | 0.00000 |
| **P. medicum 4** | 2.72578 | 0.21404 | 0.11623 | 0.03307 | 0.02587 | 0.21967 | 0.36629 | 0.00000 | 0.00000 | 0.00000 |
| **P. medicum 5** | 2.64498 | 0.03491 | 0.10800 | 0.08311 | 0.02876 | 0.29267 | 0.24738 | 0.00000 | 0.00000 | 0.00000 |
| **P. medicum 6** | 4.33553 | 0.03744 | 0.04678 | 0.00840 | 0.02400 | 0.30075 | 0.19426 | 0.00000 | 0.00000 | 0.00000 |
| **P. wawrae 1** | 0.12767 | 0.06927 | 0.00453 | 0.00216 | 0.02795 | 0.05828 | 0.29353 | 0.16298 | 2.49640 | 0.42866 |
| **P. wawrae 2** | 0.02853 | 0.04428 | 0.00185 | 0.00049 | 0.07408 | 0.20767 | 0.18438 | 0.45296 | 0.82306 | 0.50317 |
| **P. wawrae 3** | 0.07852 | 0.05299 | 0.00000 | 0.00000 | 0.00000 | 0.06593 | 0.27225 | 0.12957 | 1.96312 | 0.36019 |
| **P. wawrae 4** | 0.01217 | 0.05814 | 0.01592 | 0.00723 | 0.03724 | 0.06605 | 0.23428 | 0.27442 | 0.94208 | 0.00000 |
| **P. wawrae 5** | 0.07764 | 0.04421 | 0.00877 | 0.00143 | 0.02054 | 0.02632 | 0.10574 | 0.33113 | 1.28823 | 0.00000 |
| **P. harry-smithii 1** | 0.11347 | 0.21858 | 0.46656 | 0.28746 | 0.03187 | 0.07546 | 0.54469 | 0.23687 | 0.01818 | 1.86969 |
| **P. harry-smithii 2** | 0.02943 | 0.26763 | 0.23501 | 0.14382 | 0.00000 | 0.04504 | 1.14489 | 0.09887 | 0.07366 | 1.33481 |
| **P. harry-smithii 3** | 0.09890 | 0.08869 | 0.42886 | 0.26407 | 0.04183 | 0.05359 | 0.39285 | 0.65419 | 0.03233 | 2.77028 |
| **P. harry-smithii 4** | 0.00000 | 0.06135 | 0.50683 | 0.00815 | 0.03265 | 0.13974 | 0.59583 | 2.75391 | 0.02139 | 1.59341 |
| **P. harry-smithii 5** | 0.01108 | 0.32174 | 0.27862 | 0.01796 | 0.02475 | 0.06230 | 1.30977 | 1.90446 | 0.10330 | 1.16382 |
| **P. harry-smithii 6** | 0.11679 | 0.08821 | 0.45144 | 0.00000 | 0.03109 | 0.08446 | 0.42905 | 2.76620 | 0.04851 | 1.43906 |


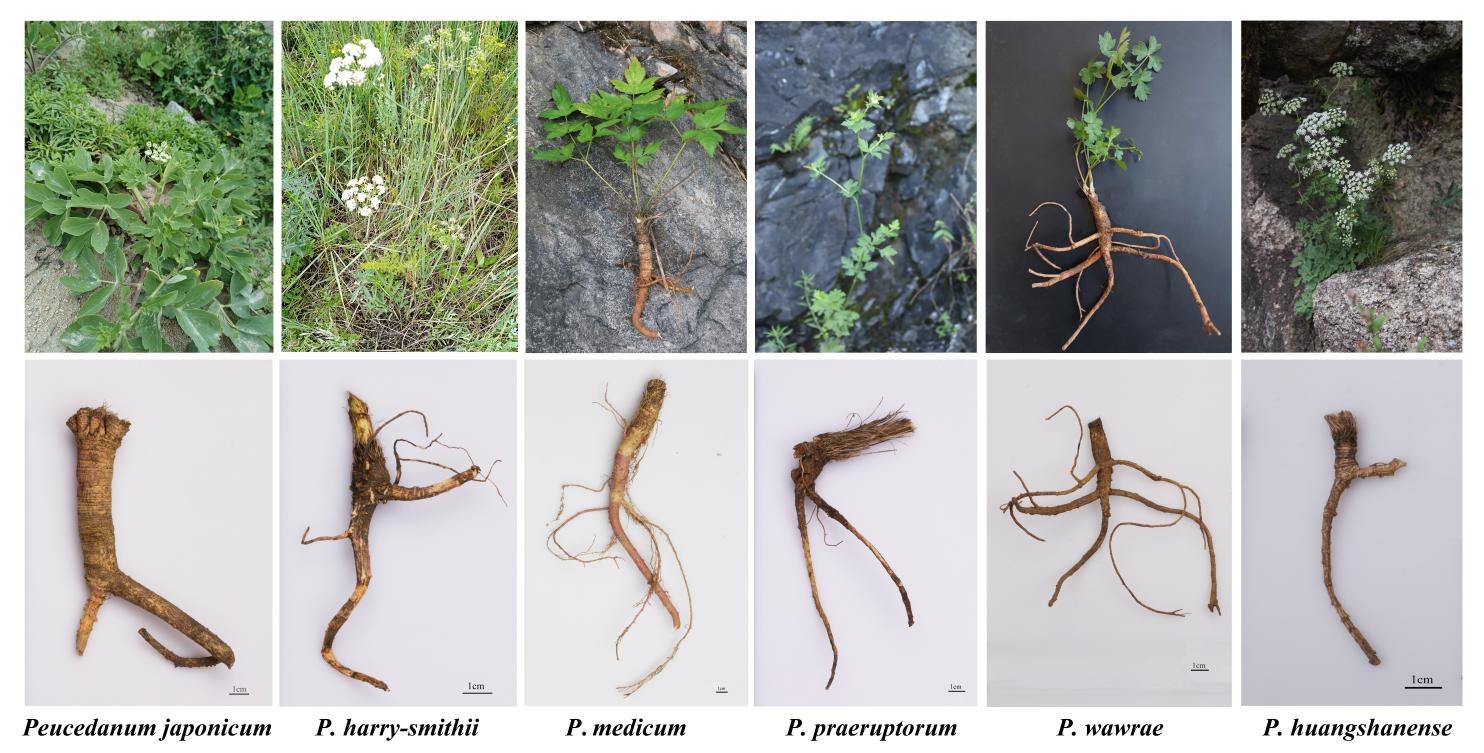


**Supplementary Figure 1** | Plants and the underground part of *P. japonicum*, *P. harry-smithii*, *P. medicum*, *P. praeruptorim*, and *P. huangshense*.


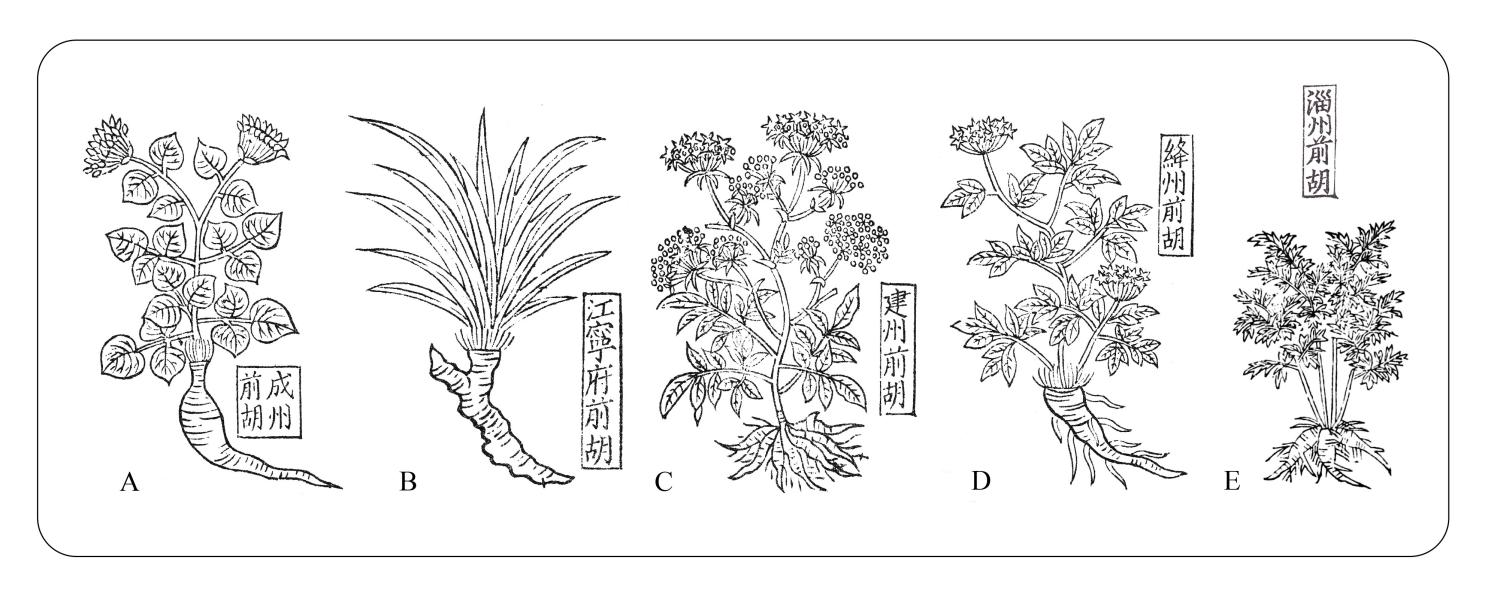


**Supplementary Figure 2** | Illustrations of Qianhu in *Ben Cao Tu Jing.* (A) Chengzhou Qianhu, (B) Jiangning Prefecture Qianhu, (C) Jianzhou Qianhu, (D) JiangZhou Qianhu, (E) Zizhou Qianhu.


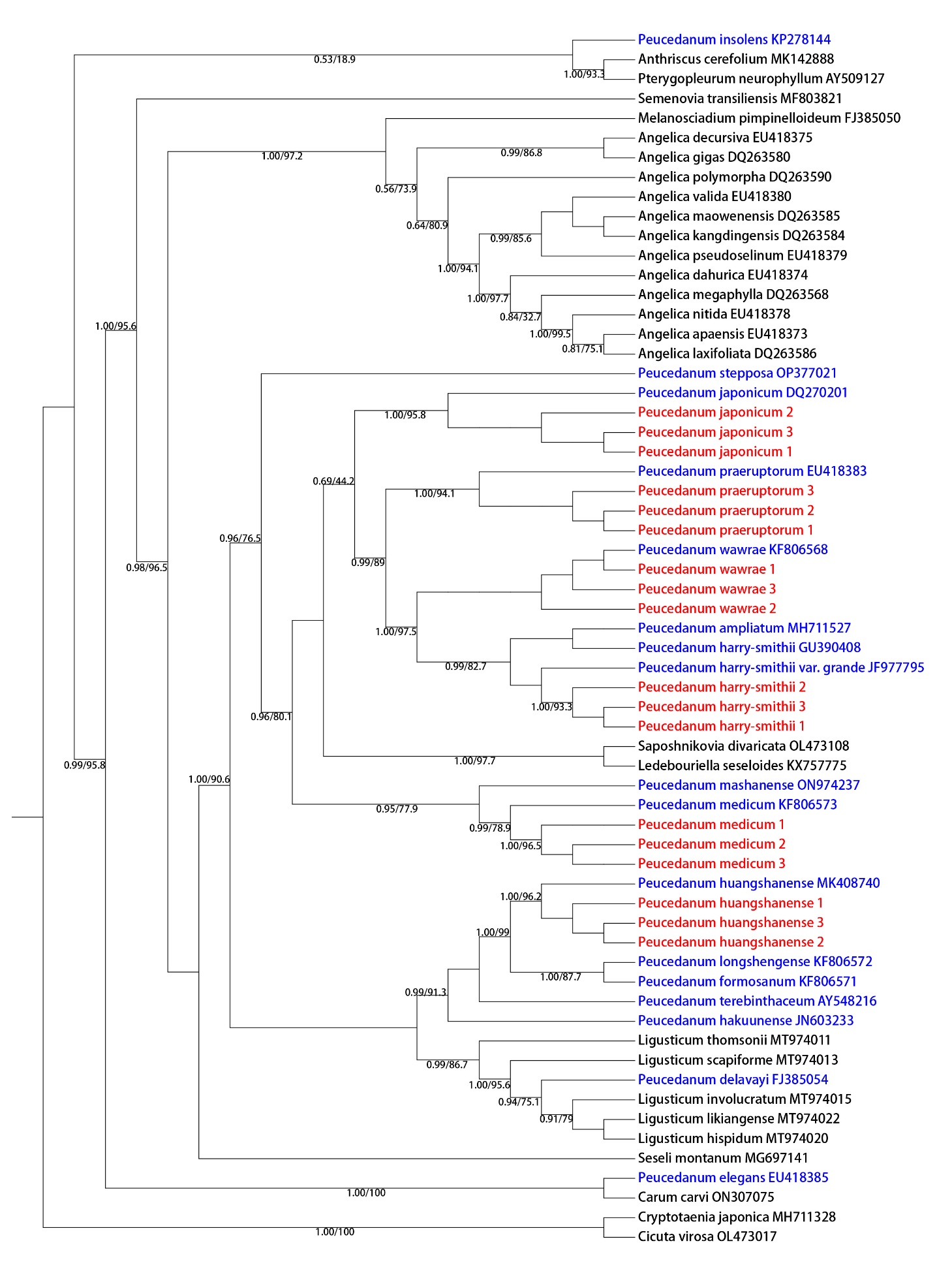


**Supplementary Figure 3** | Phylogenetic relationships inferred from maximum likelihood (ML) and Bayesian inference (BI) analyses based on ITS secquences. Numbers represent Bayesian posterior probabilities (PP) and maximum likelihood bootstrap values (BS).


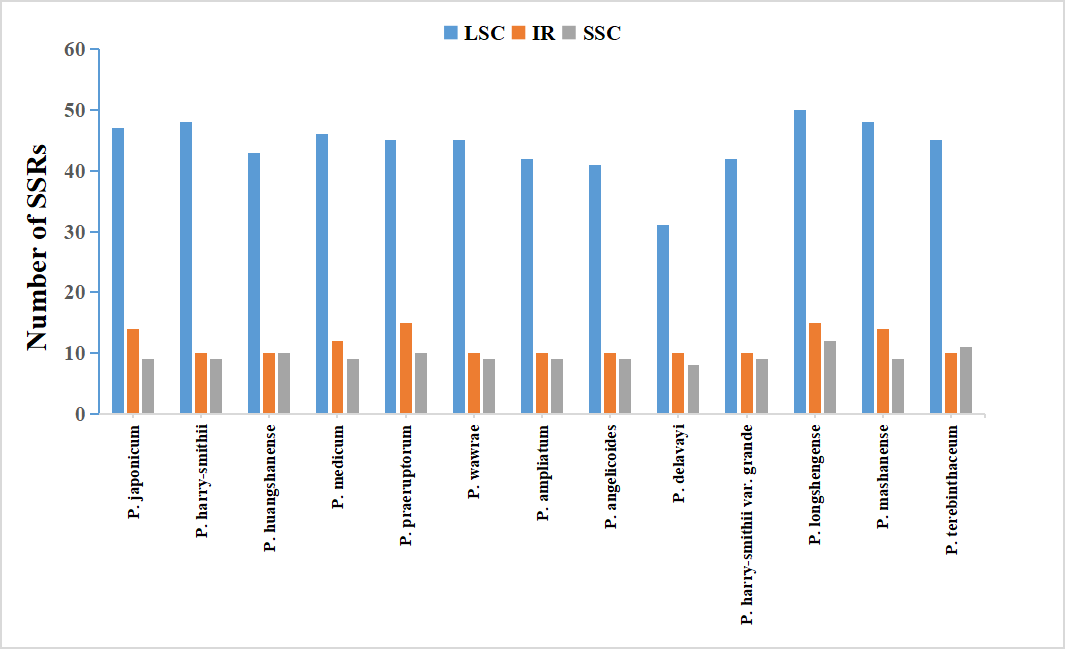


A


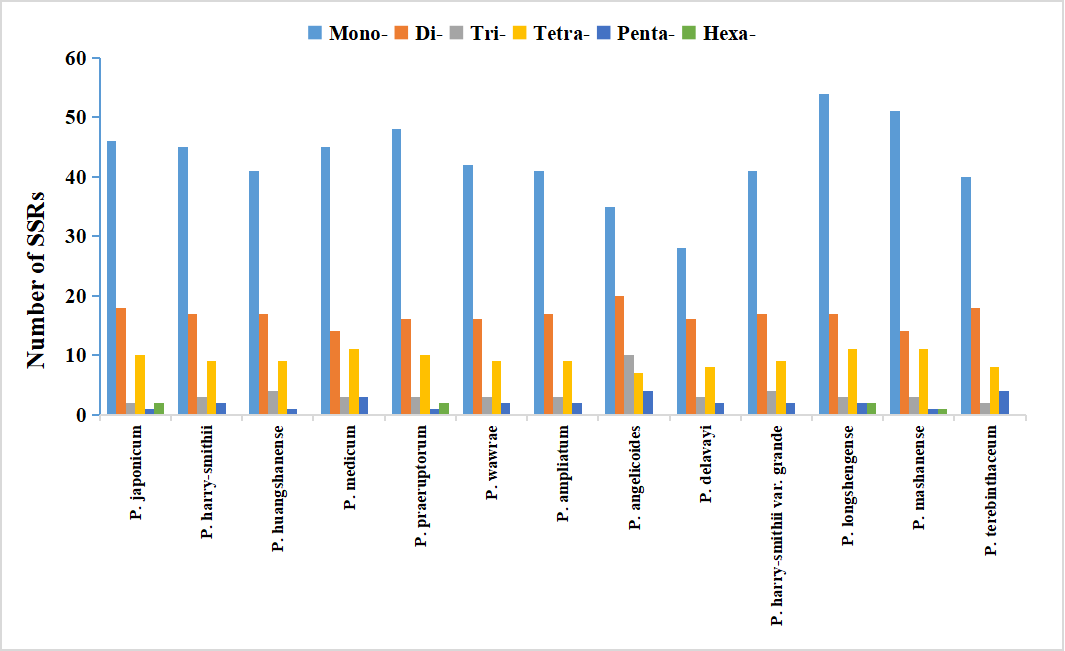


B

**Supplementary Figure 4 |** Analyses of simple sequence repeats (SSRs) in thirteen *Peucedanum* chloroplast genome (A) presence of SSRs in LSC, SSC, and IR, (B) numbers of different repeat types.


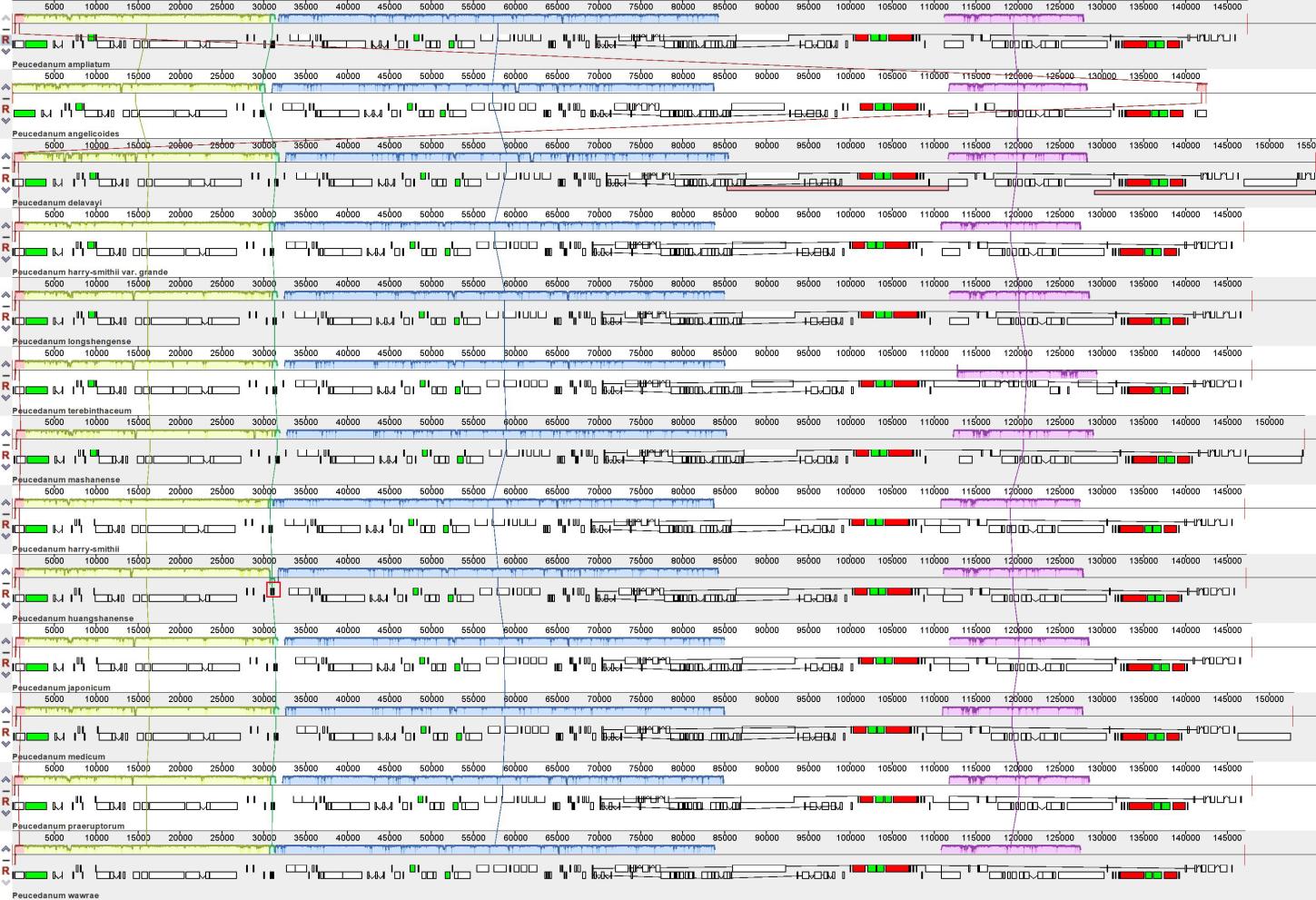


**Supplementary Figure 5** | Mauve alignment of thirteen *Peucedanum* chloroplast genomes. Local collinear blocks within each alignment are represented by blocks of the same color connected with lines. The red boxes are the inversion of the *trnY-trnD-trnE* gene of *P. huangshanense*.


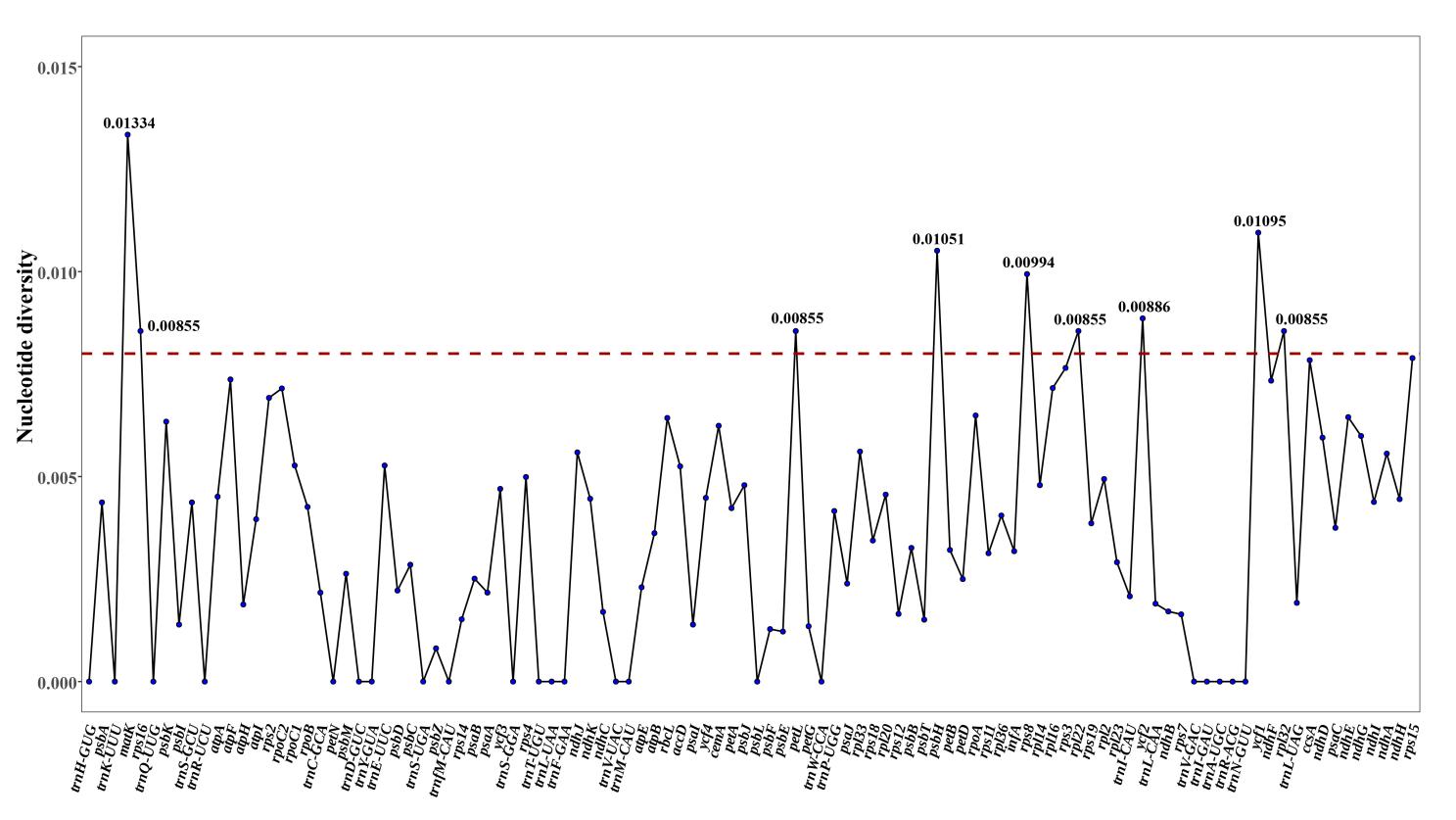


A


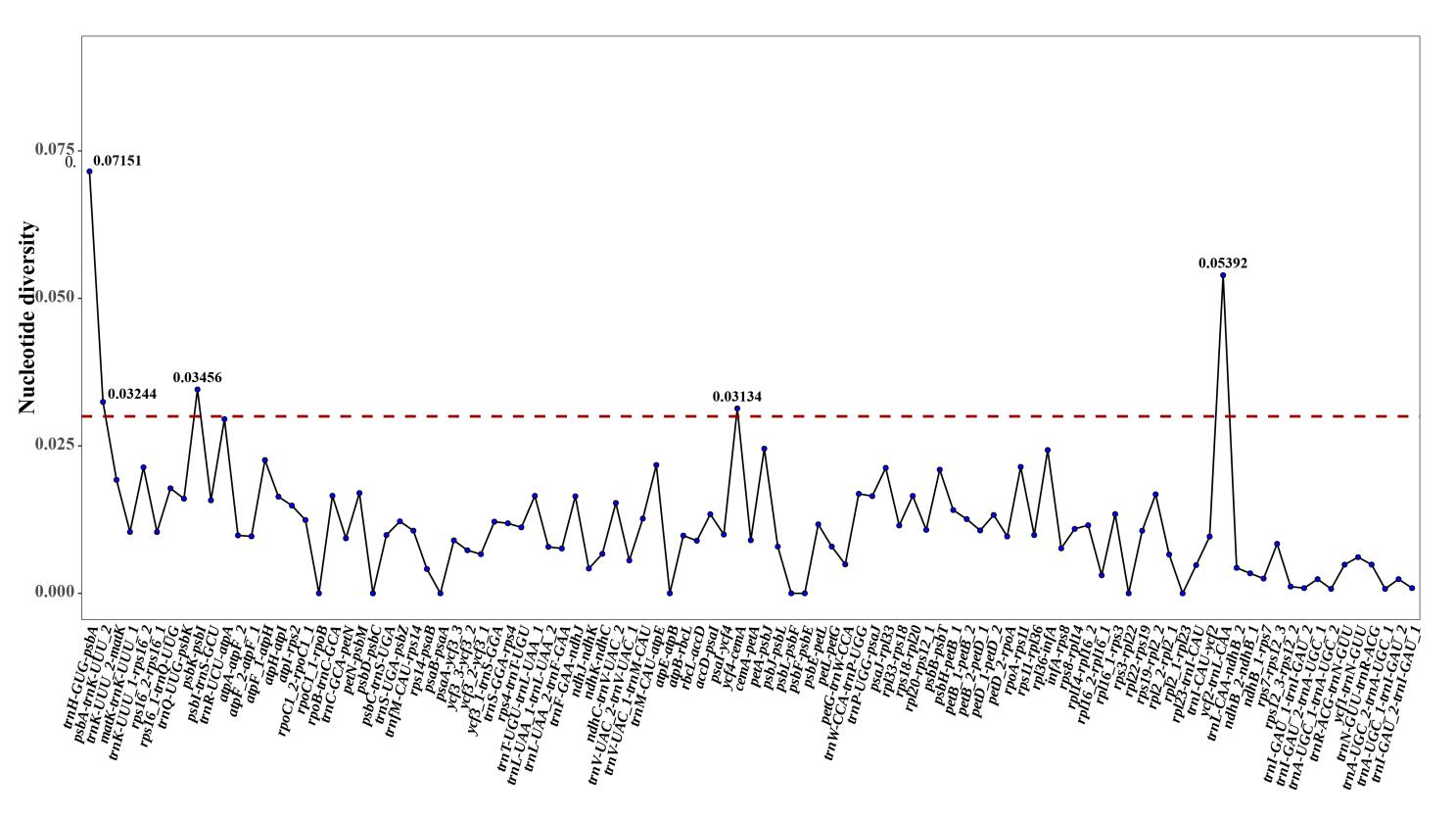


B

**Supplementary Figure 6** | Sliding-window analysis of the chloroplast genomes of thirteen *Peucedanum*. (A) Protein coding genes, (B) Non-coding and intron regions. Window length: 800bp; step size: 200bp. X-axis: position of the midpoint of a window. Y-axis: nucleotide diversity of each window
